# Organic–Inorganic Hybrid Glasses of Atomically Precise Nanoclusters

**DOI:** 10.1021/jacs.3c12296

**Published:** 2024-03-04

**Authors:** Chunwei Dong, Xin Song, Bashir E. Hasanov, Youyou Yuan, Luis Gutiérrez-Arzaluz, Peng Yuan, Saidkhodzha Nematulloev, Mehmet Bayindir, Omar F. Mohammed, Osman M. Bakr

**Affiliations:** † KAUST Catalysis Center (KCC), Division of Physical Sciences and Engineering, King Abdullah University of Science and Technology (KAUST), Thuwal 23955-6900, Saudi Arabia; ‡ Advanced Membranes and Porous Materials Center (AMPMC), and KAUST Catalysis Center (KCC), Physical Sciences and Engineering Division, King Abdullah University of Science and Technology (KAUST), Thuwal 23955-6900, Saudi Arabia; § Core Laboratories, King Abdullah University of Science and Technology (KAUST), Thuwal 23955-6900, Saudi Arabia; ∥ Center for Hybrid Nanostructures, University of Hamburg, 22761 Hamburg, Germany

## Abstract

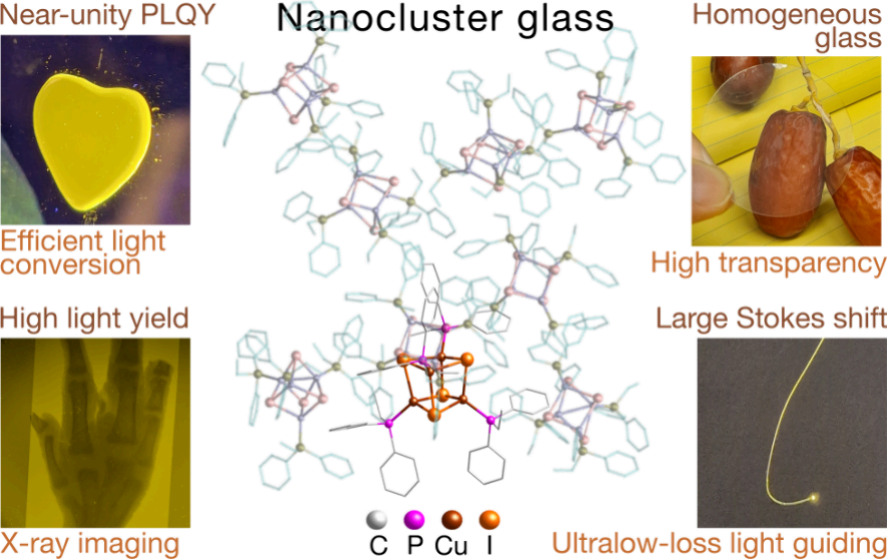

Organic–inorganic atomically precise nanoclusters provide indispensable building blocks for establishing structure–property links in hybrid condensed matter. However, robust glasses of ligand-protected nanocluster solids have yet to be demonstrated. Herein, we show [Cu_4_I_4_(PR_3_)_4_] cubane nanoclusters coordinated by phosphine ligands (PR_3_) form robust melt-quenched glasses in air with reversible crystal–liquid–glass transitions. Protective phosphine ligands critically influence the glass formation mechanism, modulating the glasses’ physical properties. A hybrid glass utilizing ethyldiphenylphosphine-based nanoclusters, [Cu_4_I_4_(PPh_2_Et)_4_], exhibits superb optical properties, including >90% transmission in both visible and near-infrared wavelengths, negligible self-absorption, near-unity quantum yield, and high light yield. Experimental and theoretical analyses demonstrate the structural integrity of the [Cu_4_I_4_(PPh_2_Et)_4_] nanocluster, i.e., iodine-bridged tetranuclear cubane, has been fully preserved in the glass state. The strong internanocluster CH−π interactions found in the [Cu_4_I_4_(PPh_2_Et)_4_] glass and subsequently reduced structural vibration account for its enhanced luminescence properties. Moreover, this highly transparent glass enables performant X-ray imaging and low-loss waveguiding in fibers drawn above the glass transition. The discovery of “nanocluster glass” opens avenues for unraveling glass formation mechanisms and designing novel luminescent glasses of well-defined building blocks for advanced photonics.

## Introduction

Glass is defined as a nonequilibrium, noncrystalline condensed matter state exhibiting a glass transition phenomenon.^1^ As a unique state of matter, glass has played a significant role in the rise of civilization and technology with its three traditional categories: inorganic, organic, and metallic glasses.^2−6^ More recently, inorganic–organic hybrid glasses have emerged as a promising family of materials, attracting considerable research interest.^7−12^ Hybrid glasses contain both inorganic and organic components that are typically connected via coordination bonds, resulting in diverse complementary advantages over their crystalline counterparts. For instance, metal–organic framework and coordination polymers—presently the main members of the hybrid glass family—exhibit multifunctional properties, such as gas permeability, thermoelectricity, luminescence, and ion conduction.^13−17^ Glasses based on hybrid metal halides have also been demonstrated, displaying intriguing optical and thermoelectric properties.^18−22^ Although inorganic–organic hybrid glasses have ignited a new field of material science, such glasses are typically susceptible to thermal decomposition or structural degradation more than their crystalline counterparts.^16−18^ As a result, a reversible crystal–liquid–glass transition with good stability, which is a prerequisite for practical implementation, has rarely been achieved for these glasses.^23,24^ Therefore, further exploration and extension of the glass family are still of significance for fundamental research and potential industrial application.

Unlike crystalline materials, amorphous glasses lack long-range periodicity, hindering atomic structure determination.^25,26^ Nevertheless, decades of dedicated research employing sophisticated modern techniques for structural characterization and simulation have led to a widely acknowledged consensus for ubiquitous structural ordering on the atomic level in amorphous glasses,^27−29^ along with the establishment of various structural models.^30−34^ One such model that has garnered broad recognition is the “solute-centered cluster” model, in which a series of interconnected clusters are organized within a medium range (up to ∼1 nm).^35,36^ The model is capable of reproducing the experimental data and accounts for the short-range order (SRO) and even medium-range order (MRO) in glasses.^37^ Nevertheless, the building blocks in the model, i.e., a variety of clusters, remain elusive because the clusters’ structure is parametrically adjusted and lacks direct experimental characterizations. Then, a fundamental query to be tackled involves whether we can identify a glass that is entirely populated by ligand-protected nanoclusters with an atomically precise structure. Identifying a glass comprised solely of such ligand-protected nanoclusters would reveal more detailed structural insights, shedding light on amorphous materials’ nature and structure–property links in glasses.^38−42^ Here, we report such a melt-quenched nanocluster glass composed of only ligand-protected nanoclusters with atomically precise structure.

Tetranuclear [Cu_4_I_4_L_4_] cubane-type nanoclusters with N- or P-donor ligands have attracted extensive interest because of their rich photophysical properties.^43,44^ Two distinct high energy (HE) and low energy (LE) emission bands are responsible for their thermochromic luminescence,^45^ which is highly dependent on the nanocluster’s polymorphism. Until now, the study of [Cu_4_I_4_L_4_] nanocluster has mainly been directed at the crystalline state, while other polymorphs such as amorphous aggregates are still desirable for their remarkable luminescence properties.^46^ We reasoned that the atomically precise structure of the [Cu_4_I_4_L_4_] nanocluster coupled with its exceptional stability and versatile functionality renders it a potentially optimal foundational unit for innovative glasses. Hence, we investigated an analogous family of [Cu_4_I_4_L_4_] nanoclusters for their glass-forming potential. We uncovered a reversible crystal–liquid–glass transition for [Cu_4_I_4_(PR_3_)_4_] cubane nanoclusters, where PR_3_ is a phosphine. The glasses are robust and reproducible through a melt-quenching process under ambient conditions, while the structural integrity of the nanocluster unit remains intact in the glass state. Taking [Cu_4_I_4_(PPh_2_Et)_4_] (where PPh_2_Et is ethyldiphenylphosphine) as a prime example, its glass phase shows remarkable photoluminescence properties, >90% transmittance over the entire visible spectrum due to its vanishingly small self-absorption and optical scattering, and has relatively high X-ray attenuation efficiency with high light yield. This combination of properties of [Cu_4_I_4_(PPh_2_Et)_4_] glass yields a material with exceptional promise for high-energy radiation scintillation and photonic applications.

## Results and Discussion

**Figure 1:**
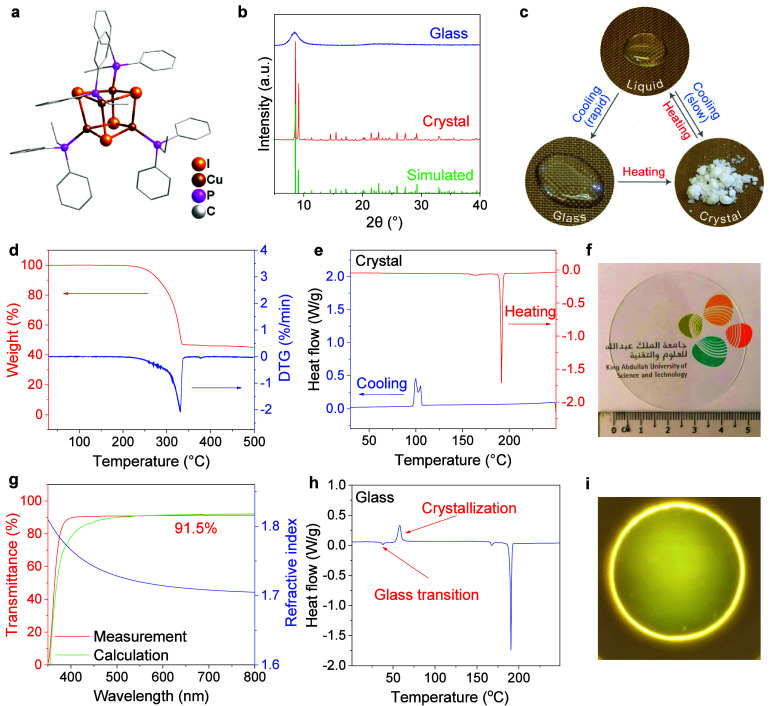
Reversible phase transitions of [Cu_4_I_4_(PPh_2_Et)_4_] nanocluster. (a) Crystal structure of [Cu_4_I_4_(PPh_2_Et)_4_] nanocluster. (b) Powder X-ray diffraction patterns of [Cu_4_I_4_(PPh_2_Et)_4_] crystal and glass. (c) Schematic illustration of the phase transitions between crystal, liquid, and glass of [Cu_4_I_4_(PPh_2_Et)_4_]. (d) Thermogravimetric (TG) analysis of [Cu_4_I_4_(PPh_2_Et)_4_] crystal. Differential scanning calorimetry traces for [Cu_4_I_4_(PPh_2_Et)_4_] (e) crystal and (h) glass with a heating/cooling rate of 5 °C/min. (g) Measured and calculated transmission spectra, and refractive index of [Cu_4_I_4_(PPh_2_Et)_4_] glass. Photographs of [Cu_4_I_4_(PPh_2_Et)_4_] glass with KAUST logo under (f) visible and (i) ultraviolet light.

[Cu_4_I_4_L_4_] nanoclusters protected by phosphine ligands have been documented to exhibit superior stability compared to those protected by pyridine ligands.^47,48^ The heightened stability implies a strong likelihood of achieving a stable melt at high temperatures. Therefore, various phosphine ligands, including triphenylphosphine (PPh_3_), methyldiphenylphosphine (PPh_2_Me), ethyldiphenylphosphine (PPh_2_Et), propyldiphenylphosphine (PPh_2_Pr), and dimethylphenylphosphine (PPhMe_2_), were selected for the synthesis of [Cu_4_I_4_(PR_3_)_4_] nanoclusters. The crystals of [Cu_4_I_4_(PR_3_)_4_] nanoclusters were synthesized using either a facile antisolvent diffusion technique or a previously reported method (see details in Methods). Single-crystal X-ray diffraction (SCXRD) measurements revealed that each product was the classical [Cu_4_I_4_(PR_3_)_4_] nanocluster, comprising four copper atoms, four iodine atoms, and four phosphine ligands. Notably, the [Cu_4_I_4_(PPh_2_Et)_4_] and [Cu_4_I_4_(PPhMe_2_)_4_] nanoclusters have not yet been structurally characterized yet. In the subsequent sections, the primary focus will be directed toward the characterization and discussion of the [Cu_4_I_4_(PPh_2_Et)_4_] nanocluster, which exhibits exceptional optical properties among the nanoclusters in this work. As shown in Figure 1a, the [Cu_4_I_4_(PPh_2_Et)_4_] nanocluster crystallizes in the orthorhombic *Aea*2 space group (Table S1) and presents the classical structure of the [Cu_4_I_4_] cubane, where the copper atoms and iodine atoms alternatively occupy the corners of a distorted cube. Four phosphine ligands are terminally coordinated to each copper atom. In addition, the pure phase of [Cu_4_I_4_(PPh_2_Et)_4_] is demonstrated by the powder X-ray diffraction (XRD) pattern (Figure 1b). Similarly, the XRD patterns of other [Cu_4_I_4_(PR_3_)_4_] nanoclusters are in excellent agreement with the corresponding simulated patterns. This correspondence strongly indicates the presence of a singular nanocluster in the product.

Thermal analyses were first conducted to reveal the nature of the melting phenomenon of the [Cu_4_I_4_(PR_3_)_4_] nanoclusters (Figure 1c). Thermogravimetric analysis (TGA) gives a decomposition temperature (*T*_d_) of 240 °C for the [Cu_4_I_4_(PPh_2_Et)_4_] crystal (Figure 1d). The weight loss at 350 °C (53.3%) matches well with the content of ethyldiphenylphosphine (52.9%) in the nanocluster. Differential scanning calorimetry (DSC) was further carried out on the [Cu_4_I_4_(PPh_2_Et)_4_] crystal. As shown in Figure 1e, a sharp endothermic peak is identified at 191 °C, which corresponds to the melting temperature (*T*_m_) of the [Cu_4_I_4_(PPh_2_Et)_4_] crystal. A minor peak below 170 °C is ascribed to the phase transition of the nanocluster (Figure S1), which is further confirmed by the temperature-dependent XRD patterns (Figure S2). Slow cooling of the melt gives rise to an exothermic peak from 106 °C in the DSC curve, corresponding to the crystallization of the supercooled liquid. The XRD pattern of the slow-cooling sample matches well with that of the original [Cu_4_I_4_(PPh_2_Et)_4_] crystal (Figure S3), suggesting the reversible crystal–liquid–crystal transition of the nanocluster (Figure 1c). Notably, *T*_m_ is far below the *T*_d_ of the nanocluster, and the weight loss at *T*_m_ is only 0.1%. These results demonstrate that a stable melt for the [Cu_4_I_4_(PPh_2_Et)_4_] nanoclusters can be formed with a relatively low melting point and wide temperature window.

Such a stable melt opens the possibility for preparing melt-quenched glasses of [Cu_4_I_4_(PR_3_)_4_] nanoclusters. Therefore, the [Cu_4_I_4_(PPh_2_Et)_4_] crystal was heated to 210 °C under ambient conditions, giving rise to a stable melt. The high-temperature melt was subsequently quenched to room temperature by transferring it to an ice–water bath or a cold plate (Figure 1c). In contrast to the slow cooling process, such fast cooling of the melt resulted in a homogeneous transparent glass (Figure 1f). Because of the highly uniform composition and substantial reduction of light scattering, the light transmission of the glass is Fresnel-reflection-limited and is higher than 90% in the visible wavelengths (Figure 1g). Spectroscopic ellipsometry was used to determine the optical constants of the glass, yielding a refractive index of 1.714 at 600 nm wavelength (Figure S4). The simulated transmission spectrum matches the measured spectrum. The hardness of bulk [Cu_4_I_4_(PPh_2_Et)_4_] glass was determined to be 0.085 ± 0.001 GPa through nanoindentation (Figure S5). Thus, [Cu_4_I_4_(PPh_2_Et)_4_] glass is softer than the typical inorganic–organic hybrid glasses,^9,49^ which could be attributed to the relatively weak van der Waals’ force between the nanoclusters rather than the strong coordinative or covalent bond in the glass framework. The XRD pattern reveals that melt-quenched [Cu_4_I_4_(PPh_2_Et)_4_] is an amorphous phase (Figure 1b). The broad peak at 8.4° is the feature of the first sharp diffraction peak (FSDP) in amorphous glasses.^19^ DSC measurements give a glass transition temperature (*T*_g_) at 36 °C for [Cu_4_I_4_(PPh_2_Et)_4_] glass (Figure 1h). The value of *T*_g_ to *T*_m_ (*T*_g_/*T*_m_ = 0.67) is consistent with the empirical “*T*_g_/*T*_m_ ∼ 2/3” law,^18^ indicating a reasonable glass-forming ability for the [Cu_4_I_4_(PPh_2_Et)_4_] nanocluster.^8^ In addition to the melting peak at 191 °C, an exothermic peak appears from 54 °C in the DSC curve of the [Cu_4_I_4_(PPh_2_Et)_4_] glass, corresponding to the crystallization process of the glass. In addition, the peak below 170 °C due to the phase transformation is also observed. XRD measurements further suggest that [Cu_4_I_4_(PPh_2_Et)_4_] glass would transform into the original crystalline phase via heat treatment (Figure S6). Thus, the [Cu_4_I_4_(PPh_2_Et)_4_] nanocluster demonstrates a reversible transition between glass, crystal, and liquid states (Figure 1c).

Moreover, it was observed that the glass formation phenomenon is universal among the [Cu_4_I_4_(PR_3_)_4_] nanoclusters. As shown in Figures S7–S10, other [Cu_4_I_4_(PR_3_)_4_] nanoclusters also demonstrate a stable melt characterized by a sharp endothermic peak preceding the decomposition. In conjunction with [Cu_4_I_4_(PPh_2_Et)_4_], the glasses derived from other [Cu_4_I_4_(PR_3_)_4_] nanoclusters can also be synthesized by using a similar methodology. As shown in Table 1, the thermal properties of the [Cu_4_I_4_(PR_3_)_4_] glasses are highly dependent on the protective phosphine ligand. The melting points range from 270 °C for the [Cu_4_I_4_(PPh_3_)_4_] nanocluster to 136 °C for the [Cu_4_I_4_(PPhMe_2_)_4_] nanocluster, while [Cu_4_I_4_(PPh_2_Me)_4_], [Cu_4_I_4_(PPh_2_Et)_4_], and [Cu_4_I_4_(PPh_2_Pr)_4_] nanoclusters exhibit a moderate melting point (187–210 °C). PPh_3_ has three phenyl substituents, imparting the largest rigidity and bulk among these ligands. Because of the high melting point, coupled with a decomposition temperature of around 280 °C, the suitability for glass formation is limited for the [Cu_4_I_4_(PPh_3_)_4_] nanocluster. On the other hand, PPh_2_Me, PPh_2_Et, and PPh_2_Pr, each featuring one alkyl and two phenyl substituents, offer more flexibility to the ligands. This increased flexibility results in the nanoclusters protected by these ligands having much lower melting points and a wide temperature window for the melt. Furthermore, PPhMe_2_, with two alkyl and one phenyl substituents, is even more flexible, leading to the [Cu_4_I_4_(PPhMe_2_)_4_] nanocluster having the lowest melting point of 136 °C. Hence, the phosphine ligand with a flexible alkyl substituent facilitates the melting process and subsequent glass formation of the nanocluster. In sharp contrast to [Cu_4_I_4_(PR_3_)_4_] nanoclusters, the [Cu_4_I_4_Py_4_] nanocluster protected by pyridine ligand does not show the melt phenomenon.^47^ As shown in Figure S11, the [Cu_4_I_4_Py_4_] nanocluster begins to show decomposition at 80 °C, with no discernible melting peak evident in the DSC curve. We speculate that the disparity in thermal stability is due to variations in the coordination ability between the ligand and copper atom. Therefore, the thermal stability of the nanocluster plays an essential role in the melting process. Notably, all [Cu_4_I_4_(PR_3_)_4_] glasses exhibit a reversible glass–crystal–liquid transition. Such a transition underscores the ability to revert the crystal structure from the glass state, implying a parallel between the local structures of the crystalline and glass states. In essence, the fabrication of [Cu_4_I_4_(PR_3_)_4_] glass is universally applicable across various phosphine ligands via a robust melt-quenching procedure under mild conditions.

**Table 1:** Summary of the Properties for [Cu_4_I_4_(PR_3_)_4_] Nanoclusters

				Emission (nm)		PLQY (%)	
Nanoclusters	*T*_m_ (°C)	*T*_*x*_ (°C) Melt/Glass	*T*_g_ (°C)	Crystal	Glass	Crystal	Glass
[Cu_4_I_4_(PPh_2_Et)_4_]	191	106/54	36	610	585	48	97
[Cu_4_I_4_(PPh_2_Me)_4_]	210	105/63	35	580	610	94	67
[Cu_4_I_4_(PPh_2_Pr)_4_]	187	103/57	33	555	575	77	67
[Cu_4_I_4_(PPh_3_)_4_]	270	195/118	111	545	570	87	28
[Cu_4_I_4_(PPhMe_2_)_4_]^a^	136	73/–	–	620	–	86	–

a [Cu_4_I_4_(PPhMe_2_)_4_] glass can be obtained via quenching the melt below 0 °C. The characterizations of this glass are not available because the glass phase is only available exclusively at low temperatures and will transform into a crystalline phase at room temperature.

**Figure 2:**
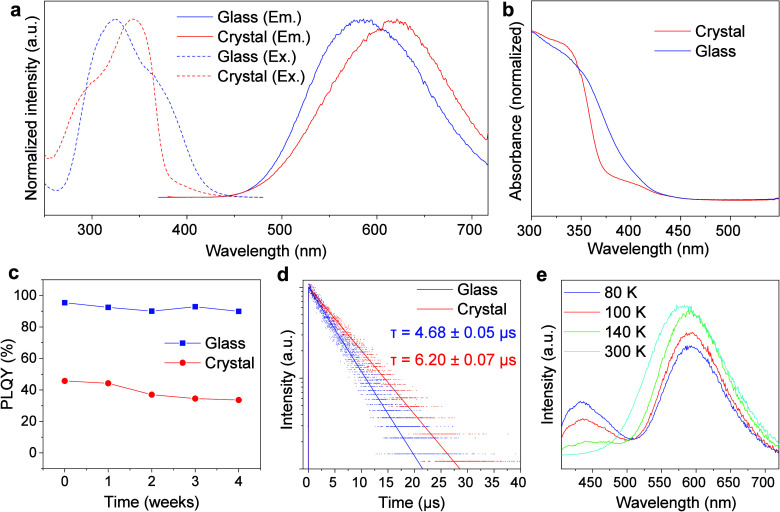
Optical properties of [Cu_4_I_4_(PPh_2_Et)_4_] glass and crystal. (a) Excitation and emission spectra, (b) UV–vis absorption spectra, (c) stability profile (time-dependent PLQY), and (d) time-resolved photoluminescence decay curves of [Cu_4_I_4_(PPh_2_Et)_4_] glass and crystal. (e) Temperature-dependent emission spectra of [Cu_4_I_4_(PPh_2_Et)_4_] glass (λ_ex_ = 365 nm).

[Cu_4_I_4_L_4_] nanoclusters are well-known for their rich luminescence properties. In this context, both the crystal and glass manifestations of [Cu_4_I_4_(PR_3_)_4_] exhibit strong emission under ultraviolet light excitation at room temperature (Figure 1i and Figure S12). Figures S7–S10 display the emission and excitation profiles of [Cu_4_I_4_(PR_3_)_4_] crystals and glasses, both of which show a broad emission band. Compared with the crystalline sample, [Cu_4_I_4_(PR_3_)_4_] glass typically manifests a red shift in emission. The sole exception is observed in the case of the [Cu_4_I_4_(PPh_2_Et)_4_] nanocluster (Figure 2a), wherein the emission peak (∼585 nm) of the glass state shifts to a shorter wavelength compared to the crystalline counterpart (∼610 nm). Notably, the [Cu_4_I_4_(PPh_2_Et)_4_] crystals, prepared by various methods including slow cooling of the melt, heating the glass, and antisolvent diffusion (solution), exhibit identical photoluminescence spectra (Figure S13). This similarity is attributed to their identical structure and polymorph, as revealed by PXRD patterns (Figure 1b, Figure S3, and Figure S6). Because the emission spectrum of the [Cu_4_I_4_L_4_] nanocluster has been correlated to the Cu–Cu distances within the [Cu_4_I_4_] core,^43^ the shift of the emission peak should be related to the structural deformation differences in [Cu_4_I_4_(PR_3_)_4_] between the crystal and glass states. In addition, similar to the crystalline state, the absorption spectrum of the [Cu_4_I_4_(PPh_2_Et)_4_] glass demonstrates an absorption onset at approximately 420 nm (Figure 2b), suggesting negligible self-absorption effects. The luminescence efficiency of the [Cu_4_I_4_(PR_3_)_4_] glass was further evaluated. In principle, the photoluminescence quantum yield (PLQY) of the glass state is expected to be lower than that of the crystalline counterpart because of the highly disordered structure and consequently severe nonradiative recombination encountered in the glass. Consequently, most of the [Cu_4_I_4_(PR_3_)_4_] glasses exhibit a PLQY lower than that of the crystals (Table 1). However, [Cu_4_I_4_(PPh_2_Et)_4_] glass defies this trend, presenting an impressive PLQY of 97% (Figure S14), which greatly surpasses the PLQY of its crystalline counterpart (48%, Figure S15). The PLQY remains as high as 90% even after 4 weeks (Figure 2c), demonstrating the good stability of [Cu_4_I_4_(PPh_2_Et)_4_] glass under ambient conditions. Moreover, the time-resolved photoluminescence experiments give a lifetime for [Cu_4_I_4_(PPh_2_Et)_4_] glass (4.68 μs) shorter than that of the crystalline sample (6.20 μs) (Figure 2d). Hence, compared with the crystalline counterpart, [Cu_4_I_4_(PPh_2_Et)_4_] glass showcases enhanced luminescence characteristics, including a near-unity PLQY, a shortened photoluminescence lifetime, Fresnel-reflection-limited transmittance, and a blue shift in the emission spectrum.

The emission and excitation spectra at varied temperatures from 80 to 300 K were recorded for [Cu_4_I_4_(PPh_2_Et)_4_] glass. As shown in Figure 2e, the broad low energy (LE) emission predominates in the spectrum at high temperatures (>200 K). By further lowering the temperature, a new high energy (HE) emission band emerges with progressively increased intensity, while the LE emission band diminishes gradually. The excitation profiles of the two emission bands are similar to maxima at ∼350 nm (Figure S16). Thus, [Cu_4_I_4_(PPh_2_Et)_4_] glass displays typical thermochromic luminescence of [Cu_4_I_4_L_4_] nanoclusters based on the thermal equilibrium between two excited states.^48^ The LE emission band can be attributed to the triplet “cluster centered” (CC) excited state of [Cu_4_I_4_L_4_] nanoclusters, involving the halide-to-metal charge transfer (XMCT) and copper-centered d → s and p transitions. In contrast, the HE emission band is “ligand-centered” with a mixed excited state of metal-to-ligand charge transfer and halide-to-ligand charge transfer (MLCT/XLCT).^50^ Overall, the luminescence properties of [Cu_4_I_4_(PPh_2_Et)_4_] glass, especially the thermochromic luminescence, are in agreement with the typical features of the common [Cu_4_I_4_L_4_] nanoclusters, suggesting the preservation of the [Cu_4_I_4_(PPh_2_Et)_4_] cubane structure in the glass state.

**Figure 3:**
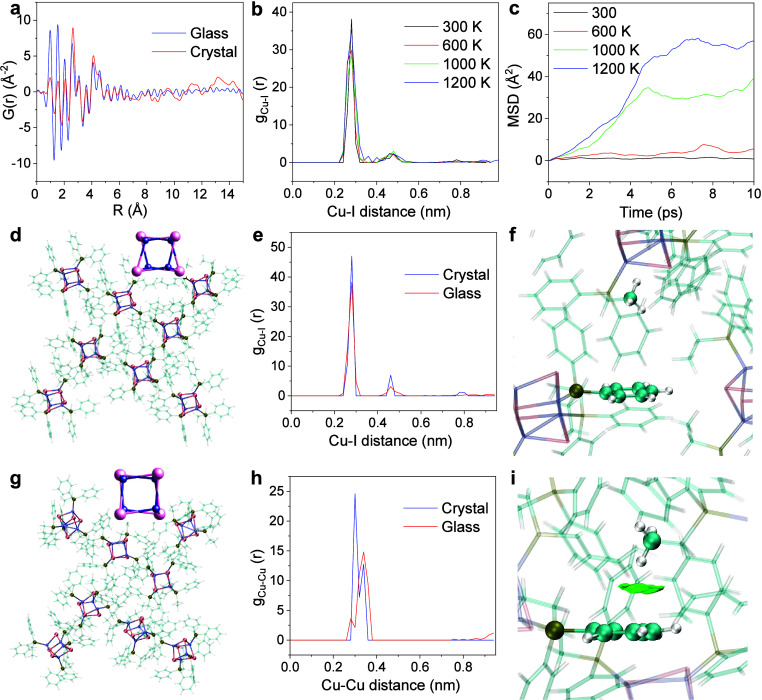
Pair distribution functions (PDFs) and ab initio molecular dynamics (AIMD) simulations of [Cu_4_I_4_(PPh_2_Et)_4_]. (a) PDFs of [Cu_4_I_4_(PPh_2_Et)_4_] crystal and glass. Evolution of the partial radial distribution function *g*_*ij*_(*r*) for (b) Cu–I distance and (c) mean square displacement (MSD) in [Cu_4_I_4_(PPh_2_Et)_4_] at varied temperatures from 300 to 1200 K based on AIMD. (d) Optimized structure of [Cu_4_I_4_(PPh_2_Et)_4_] crystal. (g) AIMD structure of [Cu_4_I_4_(PPh_2_Et)_4_] glass obtained by quenching the melt from 1000 to 273 K. Insets in (d) and (g): corresponding geometry of representative [Cu_4_I_4_] cubane in [Cu_4_I_4_(PPh_2_Et)_4_] crystal and glass. The partial radial distribution function *g*_*ij*_(*r*) for (e) Cu–I distance and (h) Cu–Cu distance in optimized [Cu_4_I_4_(PPh_2_Et)_4_] crystal and AIMD [Cu_4_I_4_(PPh_2_Et)_4_] glass. The weak interactions analyzed by the independent gradient model based on Hirshfeld partition (IGMH) for −CH_3_ to the benzene ring in (f) optimized [Cu_4_I_4_(PPh_2_Et)_4_] crystal and (i) AIMD [Cu_4_I_4_(PPh_2_Et)_4_] glass.

In addition, the nearly unchanged Fourier transform infrared (FTIR) spectra and Raman spectra (Figures S17 and S18) provide strong evidence of the same chemical components in glass and crystal, indicating a preserved coordination environment in the glass state. Nevertheless, the further structural characterization of [Cu_4_I_4_(PPh_2_Et)_4_] glass is still a great challenge because of the noncrystallinity as revealed by the XRD. The pair distribution function (PDF), which represents the probability of discovering a pair of atoms at a certain distance in the sample and yields structural information in the direct space, has been demonstrated to provide vital information on the local atomic arrangement in noncrystalline compounds. Therefore, the X-ray total scattering experiment was performed to obtain the PDF. The total scattering data of [Cu_4_I_4_(PPh_2_Et)_4_] glass give a smooth curve except for the FSDP, while the crystalline sample shows typical Bragg peaks (Figure S19). The PDF, *G*(*r*), was calculated from the total scattering data for both the crystalline and glass samples. As shown in Figure S20, the PDF oscillations of the [Cu_4_I_4_(PPh_2_Et)_4_] crystal extend over 100 Å due to the structural coherence domains with long-range order. In contrast, the structural coherence of [Cu_4_I_4_(PPh_2_Et)_4_] glass is much shorter, and *G*(*r*) is essentially featureless above 7 Å (Figure 3a). Below this distance, two samples give an identical shape of the PDF oscillations. According to the SCXRD structure of [Cu_4_I_4_(PPh_2_Et)_4_] crystal, the distance between the two farthest atoms (the copper atom and phosphorus atom in a diagonal) in the [Cu_4_I_4_P_4_] core is 6.9 Å. These results suggest that the structural unit of [Cu_4_I_4_(PR_3_)_4_] is retained in the glass state. In addition, the peaks with a short distance (<2 Å), corresponding to the C–H, C–C, and C–P bonds within the ligands, are identical for the two samples. At longer distances between 2 and 7 Å, some peaks show a minimal shift (<0.05 Å), indicating small deformation of the [Cu_4_I_4_P_4_] core in the glass state. Notably, the size of the whole [Cu_4_I_4_(PPh_2_Et)_4_] nanocluster in the crystal is approximately 14.8 Å (the two farthest carbon atoms), which is larger than the coherence domains in the glass state. This implies the irregularity of the ligand arrangements within the nanocluster, involving the orientation of the phenyl and ethyl. Hence, the structure of the [Cu_4_I_4_(PPh_2_Et)_4_] nanocluster remains preserved in the glass state, while the frameworks around copper and iodine atoms show only minor deformations.

To gain further insights into the microscopic evolution of [Cu_4_I_4_(PPh_2_Et)_4_] during the melt-quenching process, ab initio molecular dynamics (AIMD) simulations were performed using the CP2K software package. The simulated system was a 1 × 1 × 2 supercell or a 2 × 2 × 2 supercell. The simulated results based on these two supercells are consistent, as illustrated by similar data (Figures S21–S23). Because the 2 × 2 × 2 supercell system is too big to conduct the weak interaction analysis, the AIMD simulations derived from the 1 × 1 × 2 supercell will be discussed in the following. To reduce the time cost, temperatures higher than the practical *T*_m_ were chosen for the simulations, and four separate AIMD simulations were performed on the crystalline state at 300, 600, 1000, and 1200 K with a time scale of 10 ps (Figure S24). As shown in Figure S25 and Figure S26, the whole system tends to be steady after 1 ps. Hence, 10 ps is sufficient for the entire system to reach thermal equilibration for the melting and cooling process. The radial distribution functions (RDFs) and mean square displacements (MSDs) were calculated for each trajectory.

The partial RDFs, *g*_*ij*_(*r*), at varied temperatures, are shown in Figure 3 and Figure S27. As expected, the distance distributions for Cu–I, Cu–P, and Cu–Cu become broadened at higher temperatures due to the thermal vibration of the atoms. It can be seen that the Cu–I and Cu–P bond lengths are independent of temperature. In contrast, a slight change in the Cu–Cu distance is observed at different temperatures, indicating the deformation of [Cu_4_I_4_] cubane. The generalized Lindemann ratio, calculated from the width of the first peak in the partial RDFs, has been used to estimate the melting occurrence with the criterion ranging from 10% to 15%.^14^ As shown in Figure S28, the Lindemann ratios based on *g*_*ij*_(*r*) of the Cu–I and Cu–P distances are found to exceed 15% at 1000 K, illustrating the melting state of the system. In addition, the MSDs of [Cu_4_I_4_(PPh_2_Et)_4_] at 300, 600, 1000, and 1200 K are determined to be 1, 5, 39, and 57 Å^2^ at 10 ps, respectively (Figure 3c). Trajectory animations show that nanoclusters are confined within the original sites at low temperatures (300 and 600 K) because of the limited framework vibrations (Video S1). However, the thermal perturbation dramatically increases along with the apparent motion of the nanoclusters at higher temperatures (Video S1). The diffusive behavior with a remarkable increment of the displacements at 1000 and 1200 K suggests the liquid-like nature of the melt.

To shed light on the structural information on the glass state, a simulation of the melt-quenching process was performed, and the melted system was quenched from 1000 to 273 K in 10 ps (Video S2). In contrast to the ordered supercell in the crystalline state (Figure 3d), the glass state exhibits significant disorder of the internanocluster arrangement (Figure 3g) as well as a significant disorder of the ligand orientation. Despite the disorder, the structure of the [Cu_4_I_4_(PPh_2_Et)_4_] nanocluster is preserved. Moreover, the nanocluster has undergone a slight change during the melt-quenching process, leading to some deformation in the [Cu_4_I_4_] cubane (insets in Figure 3d, g). The atom–atom distances within the nanocluster are further compared for the crystalline and glass states by using the partial RDFs, *g*_*ij*_(*r*). As shown in Figure 3e, the Cu–I bond length in the glass state is identical to that of the crystalline state, which can be explained by the large tolerance of bond angles for the Cu–I ionic bond character. However, the Cu–P and Cu–Cu distances are lengthened to some extent in the glass state (Figure 3h and Figure S29).

The luminescent characteristics of [Cu_4_I_4_L_4_] cubane nanoclusters are closely linked to the [Cu_4_I_4_] core geometry, in particular, for the Cu–Cu interactions. The large Cu–Cu distance as well as the constant Cu–I bond length in the glass state leads to an expansion of the Cu_4_ tetrahedron associated with a shrinkage of the I_4_ tetrahedron. Such a change is similar to the case of the temperature dependence of the core geometry.^51^ And larger Cu_4_ tetrahedron volume (or longer Cu–Cu distance) leads to higher emission energy, which is inconsistent with the observation in [Cu_4_I_4_(PPh_2_Et)_4_] crystal and glass. According to the previous reports,^52^ the core geometry of [Cu_4_I_4_] cubane in the photoexcited T_1_ state suffers from remarkable distortions with substantially shortened Cu–Cu distance to stabilize the triplet state. Therefore, the photoexcited T_1_ state in the glass state is less stabilized than that in the crystalline state, resulting in higher energy emission, i.e., a blue shift in the emission spectrum.

To gain additional insight into the emission properties, we investigated weak interactions in both the crystalline and glass states using the independent gradient model based on Hirshfeld partition (IGMH).^81^ As shown in Figures S30–32, the [Cu_4_I_4_(PPh_2_Et)_4_] crystal shows stronger interaction within each facet in the [Cu_4_I_4_] cubane, while the interaction is much weaker in the glass state because of the longer Cu–Cu distance. Furthermore, IGMH analyses for internanocluster interaction were performed. Figure 3f and Figure S33 show the interactions between each nanocluster in the glass state, with the green area indicating strong weak interaction. This observation suggests that both −CH_2_– and −CH_3_ groups can form strong CH−π interactions with adjacent benzene rings. Conversely, such an interaction has not been found in the crystalline state (Figure 3i). Consequently, the strong CH−π interactions in the glass state enhance the rigidity of the system and suppress the structural vibration of the nanocluster. As a result, the nonradiative relaxation is impeded in the glass state, leading to a significantly higher PLQY than that in the crystalline state.

**Figure 4:**
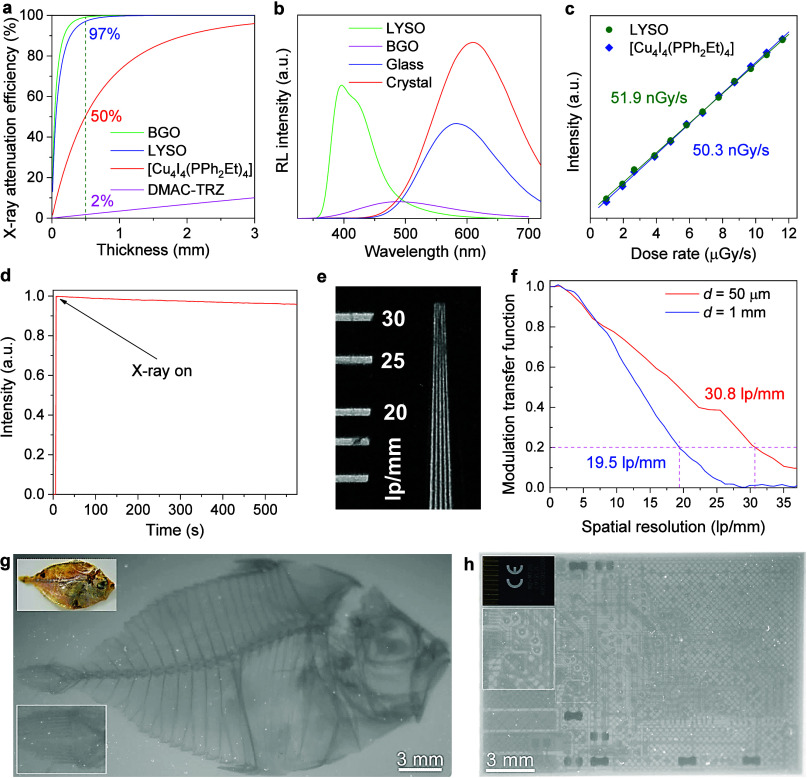
X-ray scintillation performance of [Cu_4_I_4_(PPh_2_Et)_4_] glass. (a) X-ray attenuation efficiencies of [Cu_4_I_4_(PPh_2_Et)_4_] glass, reference samples (BGO and LYSO), and organic scintillator DMAC-TRZ. (b) RL spectra of BGO, LYSO, [Cu_4_I_4_(PPh_2_Et)_4_] glass and crystal under the same X-ray irradiation (50 kV, 50 μA). (c) RL intensity dependence on the X-ray dose rate for 50 μm thick [Cu_4_I_4_(PPh_2_Et)_4_] glass and 500 μm thick LYSO reference sample. (d) RL stability of [Cu_4_I_4_(PPh_2_Et)_4_] glass profile under continuous X-ray irradiation at a dose rate of 17.9 mGy/s. (e) Spatial resolution data determined by a standard line-pair board (lp/mm) for the [Cu_4_I_4_(PPh_2_Et)_4_] glass with a thickness of 50 μm. (f) The modulation transfer function (MTF) of scintillating [Cu_4_I_4_(PPh_2_Et)_4_] glass with 50 μm and 1 mm thicknesses. X-ray images of (g) a small fish and (h) a memory card. Insets in (g) and (h): the corresponding image of the fish and the card under visible light.

Inorganic glass scintillators have received substantial research attention owing to their inherent advantages over (inorganic) single crystal scintillators, including high durability, low cost, and ease of manufacturing customizable shapes.^53−58^ However, a critical constraint in glass scintillators is the significantly diminished light yield under X-ray irradiation.^59^ The suboptimal low light yield arises from the lack of long-range order in the glass host, which hinders efficient energy transfer from the nonluminescent host to the luminescent activator.^60^ Consequently, [Cu_4_I_4_(PPh_2_Et)_4_] glass exclusively comprising a phosphor, combining the processable features of glass and the luminescence properties of the crystal, is highly desirable for scintillation application. First, the X-ray attenuation coefficients of the [Cu_4_I_4_(PPh_2_Et)_4_] nanocluster and other scintillators were calculated (Figure S34). As shown in Figure 4a, [Cu_4_I_4_(PPh_2_Et)_4_] exhibits weaker attenuation capacity than that of inorganic scintillators but much better than organic and organic–inorganic hybrid scintillators. To evaluate the light yield, commercially available BGO and LYSO scintillators were employed as the references. The radioluminescence (RL) spectra of [Cu_4_I_4_(PPh_2_Et)_4_] crystal and glass show a broad band at ∼585 nm and ∼610 nm, respectively, which are identical to their photoluminescence spectra (Figures S35, S36). The relative light yields of [Cu_4_I_4_(PPh_2_Et)_4_] crystal and glass are estimated to be ∼138000 photons/MeV and ∼74500 photons/MeV based on the X-ray attenuation efficiency normalized relative integrated area in the RL spectrum (Figure 4b). The amorphous phase has lower light yield compared to its crystalline counterpart because of (1) trapped light inside the homogeneous glass due to the total internal reflection contrary to enhanced optical scattering in the crystalline phase and (2) the inefficient energy transfer that occurs during the high-energy excitation process. The absence of long-range order in the glass structure hinders the effective propagation of excited states, leading to a limited light yield.^53,56^ To assess the sensitivity to X-ray exposure, the RL spectra of [Cu_4_I_4_(PPh_2_Et)_4_] glass at varied X-ray dose rates were recorded (Figure S38). The RL response graph was obtained by plotting the RL intensity against the radiation dose rate. As shown in Figure 4c, [Cu_4_I_4_(PPh_2_Et)_4_] glass exhibits a good linear RL response to the X-ray dose rate (0.973–11.62 μGy s^–1^), and the X-ray detection limit is calculated to be 50.3 nGy s^–1^ at a signal-to-noise ratio (SNR) of 3. The detection limit is 100 times lower than the dose rate required for regular medical diagnostics (5.5 μGy s^–1^).^61^ In addition, the RL intensity of [Cu_4_I_4_(PPh_2_Et)_4_] glass remains >95% of the initial value under continuous X-ray radiation (17.9 mGy s^–1^) for 600 s (Figure 4d), demonstrating its high radiation stability of [Cu_4_I_4_(PPh_2_Et)_4_] glass scintillator.

The glass scintillator of [Cu_4_I_4_(PPh_2_Et)_4_] exhibits remarkable transparency, resulting in minimal light scattering and reduced crosstalk within the film.^62−65^ Combined with its excellent scintillation properties, the [Cu_4_I_4_(PPh_2_Et)_4_] glass scintillator holds significant promise for achieving high-quality X-ray imaging. To assess the application prospects, X-ray imaging measurements were performed using a homemade imaging system comprising an X-ray source, a reflector, and a commercial camera. The [Cu_4_I_4_(PPh_2_Et)_4_] glass film was employed as the scintillation screen, and the initial test involved imaging a standard line-pair board to determine the spatial resolution. As shown in Figure 4e and Figure S39, the line space of 30 lp mm^–1^ is still distinguishable for a 50 μm thick scintillator screen, demonstrating an outstanding resolution of the glass scintillation screen. The modulation transfer function (MTF) based on the slanted-edge method was further studied (Figure 4f). As a result, a spatial resolution of 30.8 lp mm^–1^ was calculated at an MTF value of 0.2. It is known that a thick scintillator screen is essential for practical applications to capture more incident X-ray.^66^ Therefore, a scintillator screen with a thickness of 1 mm was further fabricated based on [Cu_4_I_4_(PPh_2_Et)_4_] glass. As shown in Figure 4f and Figure S40, the spatial resolution can still retain ∼20 lp mm^–1^, which is substantially higher than that typically observed in commercial scintillators with a similar thickness.^66,67^ The high spatial resolution combined with the large-area coverage of the glass screen greatly facilitates precise imaging for medical diagnostics and industrial detection purposes. To illustrate the potential applications in radiography, we conducted a series of imaging experiments. As shown in Figure 4g and Figure S41, X-ray irradiation clearly distinguishes the skeletal structures of a small fish and a chicken foot. Furthermore, the internal layouts of a memory card and a circuit board are readily observable, exhibiting well-defined boundaries (Figure 4h and Figure S42). The exceptional resolution and imaging performance showcased by the [Cu_4_I_4_(PPh_2_Et)_4_] glass scintillator position it as a promising candidate for future advancements in scintillator materials, especially in various fields requiring high-resolution imaging.

**Figure 5:**
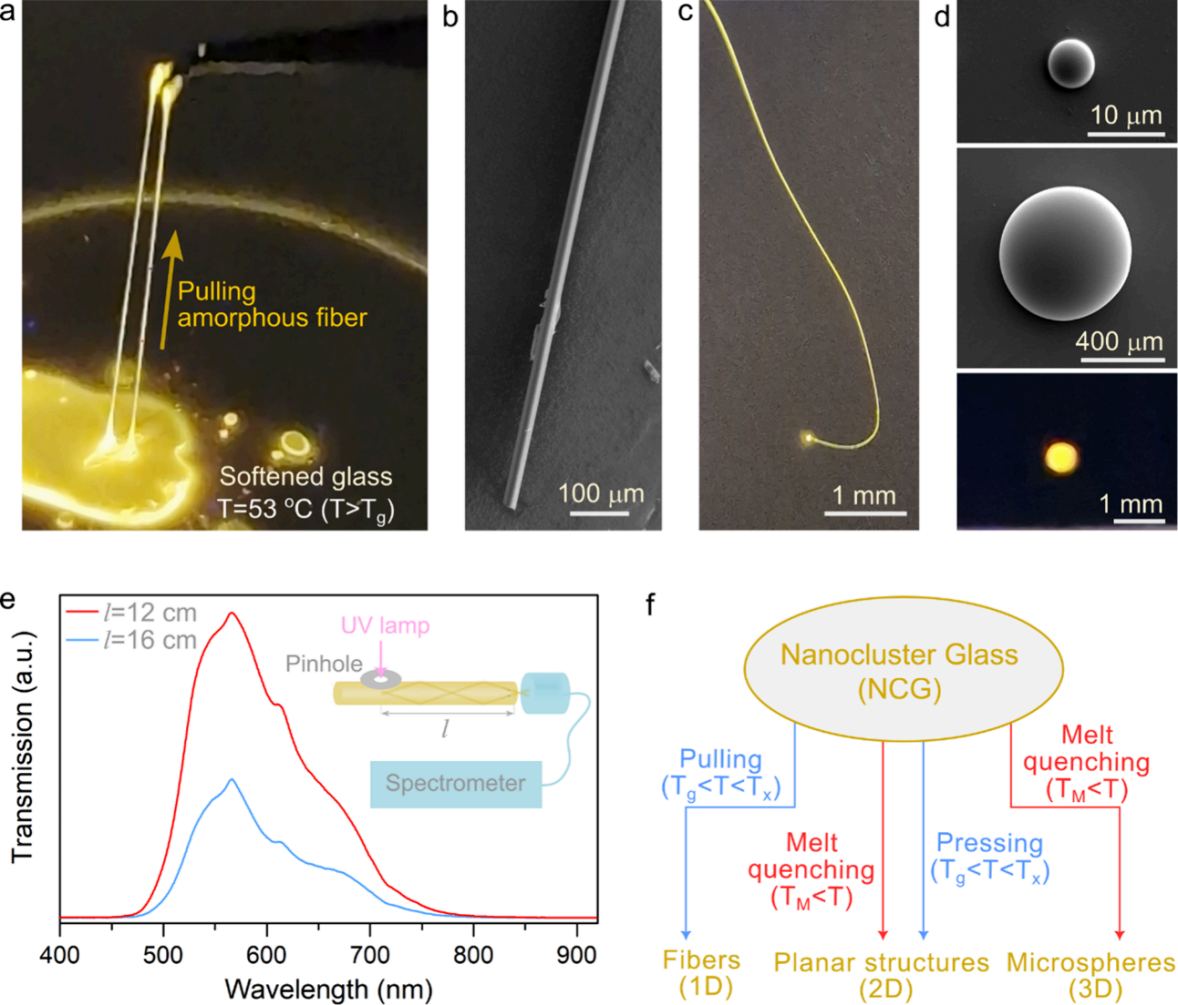
Tailoring nanocluster glass morphologies for advanced photonics. (a) Producing uniform nanocluster glass fibers from the amorphous phase by pulling with a tweezer at a temperature 53 °C. (b, c) SEM and optical images of 20 cm long nanocluster glass fiber with 45 μm diameter. Low-loss light guiding through glass fibers under UV illumination is observed. (d) Luminescent microspheres with diameters ranging from hundreds of micrometers to submicrometers are produced from melt-quenching of the nanocluster glass above its melting temperature. Bottom image: Photograph of the above microsphere under UV excitation. (e) Measured optical transmission through a glass fiber with 200 μm diameter and 30 cm total length. The propagation loss is estimated as 0.8 dB/cm at 567 nm wavelength. (f) A new class of organic–inorganic hybrid nanocluster glasses (NCG) paves the ways for the realizations of unique photonic structures.

Even though the organic–inorganic hybrid glasses promise great potential in photonic applications, the lack of transparency, self-absorption, stability, and reversible phase transitions hinder the widespread applications of these novel materials, especially in their amorphous phase. Here we demonstrated transparent, flexible, low-loss glass fibers and microresonators with ultrasmooth surfaces (Figure 5). The hybrid glass fibers were produced by pulling from the amorphous phase above its glass transition temperature (Figure 5a, Video S3). Highly uniform and flexible fibers with diameters extending from a few micrometers to several hundred micrometers were obtained, and these luminescent glass fibers exhibit strong light confinement (Figure 5b,c,e, Video S4). We achieved remarkably low propagation loss, i.e., 0.8 dB/cm at 567 nm wavelength for a 200-μm thick fiber (Figure 5e), which is significantly lower compared to previously reported organic–inorganic hybrid glass waveguides.^49,68,69^ The observation of ultralow optical loss in our nanocluster glass waveguides due to negligible self-absorption, nanocluster glass uniformity (i.e., low optical scattering), and the extremely smooth outer surface might open new avenues in advanced photonic applications. We also fabricated microspheres by melt-quenching the glass with diverse diameters (Figure 5d). Ultimately, attaining high optical transparency in visible and near-infrared (NIR) wavelengths (Figure S43), high flexibility, near unity PLQY, high light yield, and facile thermal processing (Figure 5f) of our glass will facilitate sought-after but difficult applications.

## Conclusion

In summary, we report a new family of luminescent glasses, called “nanocluster glass”, composed solely of atomically precise building blocks, i.e., [Cu_4_I_4_(PR_3_)_4_] cubane nanoclusters. The reversible crystal–liquid–glass transition with a low melting point gives rise to mild conditions for fabricating these melt-quenched glasses. Notably, the structural characteristics of the building block play a pivotal role in shaping the glass properties. Specifically, despite the significant disorder, [Cu_4_I_4_(PPh_2_Et)_4_] nanocluster glass shows enhanced optical properties over those of its crystalline counterpart, including >90% light transmission within the entire visible spectrum, negligible self-absorption, higher luminescence efficiency (a near-unity quantum yield), and high light yield, and shorter photoluminescence lifetime. The structural integrity of the tetranuclear cubane nanocluster is demonstrated to be preserved in the glass state, while the enhanced luminescence properties in glass are ascribed to the strong internanolcuster CH−π interactions and further reduced structural vibration. [Cu_4_I_4_(PPh_2_Et)_4_] glass shows a relatively high X-ray attenuation efficiency and promising X-ray scintillation performance (a light yield of ∼75000 photons/MeV; a spatial resolution of 30.8 lp mm^–1^; and a detection limit of 50.3 nGy s^–1^). Due to its ability to be processed at relatively low temperatures, typically below 200 °C, nanocluster glass can be directly deposited onto a large-area complementary metal-oxide-semiconductor (CMOS) pixelated sensor array chip. With further research and development, it holds the potential to emerge as a new generation of scintillator materials with promising prospects. We envision that the realization of glass fibers, microresonators, or periodic structures, including two-dimensional photonic crystals, might pave the way for critical applications in optics and photonics. Our work opens a new avenue for the design of novel glasses with specific structural characteristics, which is essential for correlating the structure–property relationships in this technologically critical state of matter.

## Methods

### Materials

Copper(I) iodide (99.995%), ethyldiphenylphosphine (98%), methyldiphenylphosphine (99%), dimethylphenylphosphine (97%), and triphenylphosphine (99%) were purchased from Fisher Scientific. Propyldiphenylphosphine (98%) was purchased from TCI. High-performance liquid chromatography (HPLC)-grade solvents (chloroform, dichloromethane, and diethyl ether) were purchased from VWR. All chemicals were used directly without further purification.

#### Synthesis of [Cu_4_I_4_(PPh_2_Et)_4_] Crystal

First, 0.5 mmol of copper(I) iodide and 0.5 mmol of ethyldiphenylphosphine were dissolved in 2 mL of chloroform. Then, the resultant solution was subjected to the vapor diffusion of diether ether to afford colorless crystals after several days (∼120 mg). Yield: ∼60% (based on copper).

#### Synthesis of [Cu_4_I_4_(PPh_2_Me)_4_], [Cu_4_I_4_(PPh_2_Pr)_4_], and [Cu_4_I_4_(PPhMe_2_)_4_] Crystals

First, 1 mmol of copper(I) iodide and 1 mmol of the corresponding phosphine ligands were dissolved in 2–4 mL of dichloromethane. Then, the resultant solution was subjected to the vapor diffusion of diether ether to afford colorless crystals after several days. The products for [Cu_4_I_4_(PPh_2_Me)_4_] and [Cu_4_I_4_(PPh_2_Pr)_4_] crystals were found to be identical to previous reports.^70,71^ Yield (based on copper): ∼88%, ∼75%, and ∼67% for [Cu_4_I_4_(PPh_2_Me)_4_], [Cu_4_I_4_(PPh_2_Pr)_4_], and [Cu_4_I_4_(PPhMe_2_)_4_] crystals, respectively. Cu_4_I_4_(PPh_3_)_4_] crystal was synthesized based on the reported method.^72^

#### Synthesis of [Cu_4_I_4_(PPh_2_Et)_4_] Glass

The as-prepared crystals of [Cu_4_I_4_(PPh_2_Et)_4_] were transferred to a vial or placed on a substrate (glass or plastics). The nanocluster was heated to 210 °C under ambient conditions and became a melt. After that, the nanocluster was rapidly cooled to room temperature in an ice–water bath or on a cold plate. The whole cooling process takes less than 1 min. Then homogeneous and transparent glass was obtained. Other [Cu_4_I_4_(PR_3_)_4_] glasses were obtained via a similar procedure. The [Cu_4_I_4_(PPh_3_)_4_] nanocluster was heated in a sealed vial because of its narrow melting temperature window before decomposition. The [Cu_4_I_4_(PPhMe_2_)_4_] glass was obtained via quenching the melt to ice water rather than at room temperature. Once warming the glass was warmed to room temperature, the [Cu_4_I_4_(PPhMe_2_)_4_] glass will transform into a crystal.

### Characterization

SCXRD measurements were conducted on a Bruker D8 Venture diffractometer with a SMART APEX2 area detector (Mo Kα, λ = 0.71073 Å). The X-ray crystallographic data (Table S1) for [Cu_4_I_4_(PPh_2_Et)_4_] and [Cu_4_I_4_(PPhMe_2_)_4_] have been deposited at the Cambridge Crystallographic Data Centre (CCDC), under deposition numbers 2293993 and 2293992. These data can be obtained free of charge from the CCDC via www.ccdc.cam.ac.uk. Room temperature powder X-ray diffraction measurements were performed on a Bruker D8 ADVANCE diffractometer with Cu Kα radiation (λ = 1.54 Å). Temperature-dependent X-ray diffraction measurements were performed on a similar diffractometer with a temperature-controlled system. The raw data for pair distribution function analysis were collected on a D8 discover system with a Focused Gobbel Mirror and 2D Eiger detector in a 0.5 mm capillary. The X-ray source was Mo with a wavelength of 0.71073 Å. The empty capillary pattern was also collected as the background. The PDFgetX3 component of the same program was used to correct and normalize the diffraction data. Then the data were subjected to Fourier transform to obtain PDF, G(r). G(r) gives the probability of finding a pair of atoms separated by a distance of r. The PDF data were also refined with a small box model with the TOPAS 6 PDF refinement module. Thermogravimetric analysis (TGA) was performed on a TGA-TA Discovery 5500 instrument. The heating rates for all samples were set as 10 °C min^–1^ under nitrogen flow. Differential scanning calorimetry (DSC) analysis was carried out on a DSC-TA Discovery 250 instrument. The heating or cooling rate was set as 5 °C min^–1^ under a nitrogen atmosphere. Room temperature steady-state photoluminescence spectra were recorded with a fluorescence spectrophotometer (Horiba Fluoromax-4). Photoluminescence quantum yields (PLQYs) were recorded on an Edinburgh F255 spectrophotometer. The TRPL measurements were performed in a Halcyone spectrometer (Ultrafast Systems) in the time-correlated single-photon counting configuration. The excitation wavelength was selected to be 350 nm using an optical parametric amplifier (Newport, Spectra-Physics) that was pumped with an Astrella femtosecond pulsed laser (800 nm, 150 fs, 1 kHz, Coherent). The resulting photoluminescence was collected and passed through a long-pass filter (550 nm, Newport) and focused on an optical fiber coupled to a monochromator. The detection wavelength was at the respective emission maxima and sent to a PMT detector. TCSPC histograms were fitted using the Lavenberg–Marquart algorithm as implemented in Ultrafast System software. The overall time resolution for the system was better than 150 ps. The UV–vis spectra and transmittance spectra were recorded on a Lambda 950 spectrophotometer equipped with an integrating sphere. The measurements of hardness were performed on a nanoindentation tester (NanoTest Vantage) at room temperature (23 °C).

### Determination of Optical Constants of Glass

We measured the refractive index, *n*, and extinction coefficient, *k*, using a variable angle spectroscopic ellipsometer (J. A. Woollam, M-2000 DI) at angles of incidence 65°, 70°, and 75° to increase accuracy. The measured values were simultaneously fitted with an isotropic “Cauchy” model in the range 300–1900 nm.

### Computational Details

To understand the melt-quenching process of the [Cu_4_I_4_(PPh_2_Et)_4_] nanocluster, ab initio molecular dynamics (AIMD) were performed using the CP2K software package.^73^ The simulation was conducted using a 1 × 1 × 2 supercell along the *z*-axis and a 2 × 2 × 2 supercell along all dimensions. PBE^74^ with Grimme D3 correction^75^ was used to describe the system. The Goedecker–Teter–Hutter (GTH) pseudopotentials^76,77^ and DZVP-MOLOPT-GTH basis sets^78^ were utilized to describe the molecules. The NPT ensemble has been performed at 300, 600, 1000, and 1200 K for 10 ps using Canonical sampling through velocity rescaling with the time step of 1 fs.^79^ The mean square displacements (MSDs) and radial distribution functions (RDF) were calculated during the melting simulation. The quenching process was performed by decreasing the temperature from 1000 to 273 K with a timecon of 100 for 10 ps to simulate the rapid cooling. The weak interactions were analyzed by the method of independent gradient model based on Hirshfeld partition (IGMH).^80,81^ The generalized Lindemann ratio, derived from the width of the first peak in RDF, is determined following the methodology in the previous report.^14^

### X-ray Attenuation Efficiency Calculation

The linear attenuation coefficient α(*E*) (cm^–1^) was obtained by the following formula:
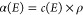
where ρ is the mass density of the scintillator, *c*(*E*) is the photon cross section function obtained from XCOM: Photon Cross Sections Database,^82^ and *E* is the corresponding X-ray photon energy (keV). The attenuation efficiency (*XAE*) for a scintillator with a thickness of *d* at a specific photon energy (*E*) is calculated from the following formula:
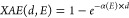
The total X-ray attenuation efficiency versus scintillator thickness for the entire X-ray spectrum (from 0 to maximum energy *E*_m_) could be obtained by the following formula:
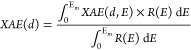
where *R*(*E*) is the energy spectrum of the X-ray source (Figure S29).

### Determination of the Light Yield

The light yields of the [Cu_4_I_4_(PPh_2_Et)_4_] crystal and glass were calculated via a reference method. Commercial BGO and LYSO scintillators with a thickness of 0.5 mm were employed as the reference samples. Both glass and crystalline samples with 0.5 mm in thickness were polished to have optically smooth surfaces. The RL spectra were recorded on a fluorescence spectrophotometer (Horiba Fluoromax-4) equipped with a photomultiplier tube (Hamamatsu, R928) and a portable X-ray source (Moxtek, Tungsten target) at an operating voltage of 50 kV and 50 μA current. The measured signals were corrected using the photomultiplier tube detector efficiency. The RL spectra of all samples were measured under identical conditions. Since the X-ray attenuation efficiency *XAE* for each material is different, the normalized spectrum was calculated using the following formula:
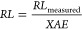
The corresponding RL photon counts were then obtained by integrating the RL spectra. The light yield of the scintillator can be calculated by the following formula
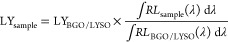
where the LY_BGO/LYSO_ is the light yield of BGO (∼8000 photons/MeV) or LYSO (∼30000 photons/MeV) and RL_sample_ and RL_BGO/LYSO_ are the normalized RL spectra of the sample and references. As a control measurement, BGO (LYSO) crystal light yield was obtained as 8200 photons/MeV (29200 photons/MeV) using LYSO (BGO) as a reference.

### Detection Limit

The RL spectra of the sample were recorded at varied X-ray dose rates. The intensity of the RL spectrum was plotted against the dose rate. The detection limit was determined as the dose rate when the signal was three times the dark noise.

### X-ray Imaging

All X-ray imaging experiments were conducted on a homemade X-ray imaging system. The system consists of an X-ray source (Moxtek, Tungsten target), a reflector, and a commercial camera (D7100, Nikon). The incident X-ray passes through the object and converts into visible light in the scintillator. A commercial camera then took a picture of the object.

## Supporting Information Available

Supporting characterizations for other nanoclusters, spectroscopic data, computational data, and crystallographic data (PDF)
Video S1: AIMD simulation trajectory at 300, 600, 1000, and 1200 K (AVI)
Video S2: AIMD simulation trajectory for the quenching process from 1000 to 273 K (AVI)
Video S3: Pulling glass fibers from amorphous phase (AVI)
Video S4: Waveguiding through hybrid glass fibers (AVI)

## Supplementary Material

ja3c12296_si_001.pdf

ja3c12296_si_002.avi

ja3c12296_si_003.avi

ja3c12296_si_004.avi

ja3c12296_si_005.avi
