# Supplementary material for: Organic–Inorganic Hybrid Glasses of Atomically Precise Nanoclusters
Source: J Am Chem Soc. 2024 Mar 4;146(11):7373–85. doi: 10.1021/jacs.3c12296 (PMC10958519; doi:10.1021/jacs.3c12296)
Supplement: Supplementary file 1 — ja3c12296_si_001.pdf [file ja3c12296_si_001.pdf]

Supporting Information

Organic-inorganic hybrid glasses of atomically precise nanoclusters

Chunwei Dong,[†,#] Xin Song,[†,#] Bashir E. Hasanov,[†] Youyou Yuan,[§] Luis Gutiérrez-Arzaluz,[‡] Peng Yuan,[†] Saidkhodzha Nematulloev,[†] Mehmet Bayindir,[‖*] Omar F. Mohammed,[‡] and Osman M. Bakr[†*]

[†]KAUST Catalysis Center (KCC), Division of Physical Sciences and Engineering, King Abdullah University of Science and Technology (KAUST), Thuwal 23955-6900, Saudi Arabia

[‡]Advanced Membranes and Porous Materials Center (AMPMC), and KAUST Catalysis Center (KCC), Physical Sciences and Engineering Division, King Abdullah University of Science and Technology (KAUST), Thuwal 23955-6900, Saudi Arabia

[§]Core Laboratories, King Abdullah University of Science and Technology (KAUST), Thuwal 23955-6900, Saudi Arabia

[‖]Center for Hybrid Nanostructures, University of Hamburg, 22761 Hamburg, Germany

[#]These authors contributed equally.

[*]Corresponding authors. E-mail: osman.bakr@kaust.edu.sa

mehmet.bayindir@uni-hamburg.de

Contents

Table S1. Crystal data and structure refinement for [$Cu_4I_4(PPh_2Et)_4$] and [$Cu_4I_4(PPhMe_2)_4$].
Figure S1. Details of the minor peak below 170 °C in the DSC trace of [$Cu_4I_4(PPh_2Et)_4$] crystal.
Figure S2. Temperature-dependent powder X-ray diffraction (XRD) patterns of [$Cu_4I_4(PPh_2Et)_4$] crystal.
Figure S3. XRD pattern of [$Cu_4I_4(PPh_2Et)_4$] prepared by slow cooling from the melt.
Figure S4. Refractive index (*n*) and extinction coefficient (*k*) of [$Cu_4I_4(PPh_2Et)_4$] glass.
Figure S5. Load-displacement curves obtained in the nanoindentation experiment of bulk [$Cu_4I_4(PPh_2Et)_4$] glass.
Figure S6. XRD pattern of [$Cu_4I_4(PPh_2Et)_4$] prepared by heating the glass at 100 °C for 1 h.
Figure S7. Characterizations of [$Cu_4I_4(PPh_2Me)_4$] crystal and glass.
Figure S8. Characterizations of [$Cu_4I_4(PPh_2Pr)_4$] crystal and glass.
Figure S9. Characterizations of [$Cu_4I_4(PPh_2Pr)_4$] crystal and glass.
Figure S10. Characterizations of [$Cu_4I_4(PPhMe_2)_4$] crystal.
Figure S11. Characterizations of [$Cu_4I_4Py_4$] nanocluster.
Figure S12. Photographs of [$Cu_4I_4(PPh_2Et)_4$] crystal under daylight and UV light.
Figure S13. Photoluminescence spectra of [$Cu_4I_4(PPh_2Et)_4$] crystals prepared by different methods.
Figure S14. Photoluminescence quantum yield (PLQY) data of [$Cu_4I_4(PPh_2Et)_4$] glass.
Figure S15. Photoluminescence quantum yield (PLQY) data of [$Cu_4I_4(PPh_2Et)_4$] crystal.
Figure S16. Excitation and emission spectra of [$Cu_4I_4(PPh_2Et)_4$] glass at 80 K.
Figure S17. Fourier transform infrared (FTIR) spectra of [$Cu_4I_4(PPh_2Et)_4$] glass, crystal, and PPh_2Et.
Figure S18. Raman spectra of [$Cu_4I_4(PPh_2Et)_4$] glass and crystal.
Figure S19. The X-ray total scattering data of [$Cu_4I_4(PPh_2Et)_4$] glass and crystal. The data were collected using a molybdenum target (Mo Kα, λ = 0.71073 Å).
Figure S20. Global pair distribution functions (PDFs) of [$Cu_4I_4(PPh_2Et)_4$] crystal and glass.
Figure S21. Simulated structures of [$Cu_4I_4(PPh_2Et)_4$] crystal and glass derived from a 2x2x2 supercell.
Figure S22. AIMD simulation results derived from a 2x2x2 supercell.
Figure S23. AIMD simulation results derived from a 2x2x2 supercell.
Figure S24. Screenshots of AIMD simulation of [$Cu_4I_4(PPh_2Et)_4$] crystal at various temperatures.
Figure S25. The temperature and potential energy vs. time plots throughout the whole simulation derived from a 1x1x2 supercell.
Figure S26. The temperature and potential energy vs. time plots throughout the whole simulation derived from a 2x2x2 supercell.
Figure S27. Evolution of the partial radial distribution function $g_{ij}(r)$ for Cu–Cu distance in [$Cu_4I_4(PPh_2Et)_4$] at varied temperatures from 300 K to 1200 K based on AIMD.
Figure S28. The generalized Lindemann ratios based on (a) Cu–P and (b) Cu–I distances.
Figure S29. The partial radial distribution function $g_{ij}(r)$ for Cu–P distance in optimized [$Cu_4I_4(PPh_2Et)_4$] crystal and AIMD [$Cu_4I_4(PPh_2Et)_4$] glass.
Figure S30. Color-bar of Independent Gradient Model (IGM) analysis.
Figure S31. The weak interactions withing the [$Cu_4I_4P_4$] core of [$Cu_4I_4(PPh_2Et)_4$] crystal analyzed by

IGMH. Purple: Cu; tan; P; and pink: I. All carbon and hydrogen atoms are omitted.

Figure S32. The weak interactions withing the [$Cu_4I_4P_4$] core of [$Cu_4I_4(PPh_2Et)_4$] glass analyzed by IGMH. Purple: Cu; tan; P; and pink: I. All carbon and hydrogen atoms are omitted.

Figure S33. The weak interactions analyzed by IGMH for -CH_2- to benzene ring in AIMD [$Cu_4I_4(PPh_2Et)_4$] glass.

Figure S34. X-ray attenuation coefficient as a function of photon energy for [$Cu_4I_4(PPh_2Et)_4$], CsI, BGO, *o*-ITC, anthracene, and $(PPh_4)_2MnBr_4$.

Figure S35. Comparison of the PL and RL spectra for [$Cu_4I_4(PPh_2Et)_4$] crystal.

Figure S36. Comparison of the PL and RL spectra for [$Cu_4I_4(PPh_2Et)_4$] glass.

Figure S37. The energy spectrum of the X-ray source up to 50 keV.

Figure S38. RL spectra of [$Cu_4I_4(PPh_2Et)_4$] glass under varied X-ray dose rates.

Figure S39. The spatial resolution data of a [$Cu_4I_4(PPh_2Et)_4$] glass with a thickness of 50 μm.

Figure S40. The spatial resolution data of a [$Cu_4I_4(PPh_2Et)_4$] glass with a thickness of 1 mm.

Figure S41. X-ray images of a chicken foot.

Figure S42. X-ray image of a circuit board.

Figure S43. Transmission through [$Cu_4I_4(PPh_2Et)_4$] glass in near-infrared (NIR) wavelengths.

Video S1: AIMD simulation trajectory at 300 K, 600 K, 1000 K and 1200 K.

Video S2: AIMD simulation trajectory for the quenching process from 1000 K to 273 K.

Video S3: Pulling glass fibers from amorphous phase.

Video S4: Waveguiding through hybrid glass fibers.

Table S1. Crystal data and structure refinement for $[Cu_4I_4(PPh_2Et)_4]$ and $[Cu_4I_4(PPhMe_2)_4]$.

	$[Cu_4I_4(PPh_2Et)_4]$	$[Cu_4I_4(PPhMe_2)_4$
Empirical formula	$C_{56}H_{60}Cu_4I_4P_4$	$C_{128}H_{176}Cu_{16}I_{16}P_{16}$
Formula weight	1618.68	5257.24
Temperature/K	240	120
Crystal system	orthorhombic	monoclinic
Space group	*Aea*2	*C2/c*
a/Å	19.477(5)	25.536(2)
b/Å	19.485(5)	11.7134(8)
c/Å	15.639(4)	19.0514(16)
α/°	90	90
β/°	90	132.208(2)
γ/°	90	90
Volume/Å^3	5935(2)	4221.0(6)
Z	4	1
ρ_{calc}g/cm^3	1.812	2.068
μ/mm^{-1}	3.637	5.087
F(000)	3136	2496
Crystal size/mm^3	0.11 × 0.06 × 0.04	0.1 × 0.07 × 0.06
Radiation	Mo *K*α (λ = 0.71073)	Mo *K*α (λ = 0.71073)
2Θ range for data collection/°	4.674 to 52.818	4.088 to 55.864
Index ranges	$-24 \le h \le 24$, $-23 \le k \le 24$, $-19 \le l \le 19$	$-33 \le h \le 33$, $-15 \le k \le 15$, $-25 \le l \le 25$
Reflections collected	47261	34678
Independent reflections	6082 [R_{int} = 0.0507, R_{sigma} = 0.0275]	5016 [R_{int} = 0.0346, R_{sigma} = 0.0218]
Data/restraints/parameters	6082/176/321	5016/0/203
Goodness-of-fit on F^2	1.098	1.203
Final R indexes [I ≥ 2σ (I)]	R_1 = 0.0247, wR_2 = 0.0449	R_1 = 0.0298, wR_2 = 0.0742
Final R indexes [all data]	R_1 = 0.0312, wR_2 = 0.0479	R_1 = 0.0359, wR_2 = 0.0786
Largest diff. peak/hole / e Å^{-3}	0.32/-0.35	1.65/-0.57
Flack parameter	0.010(11)	

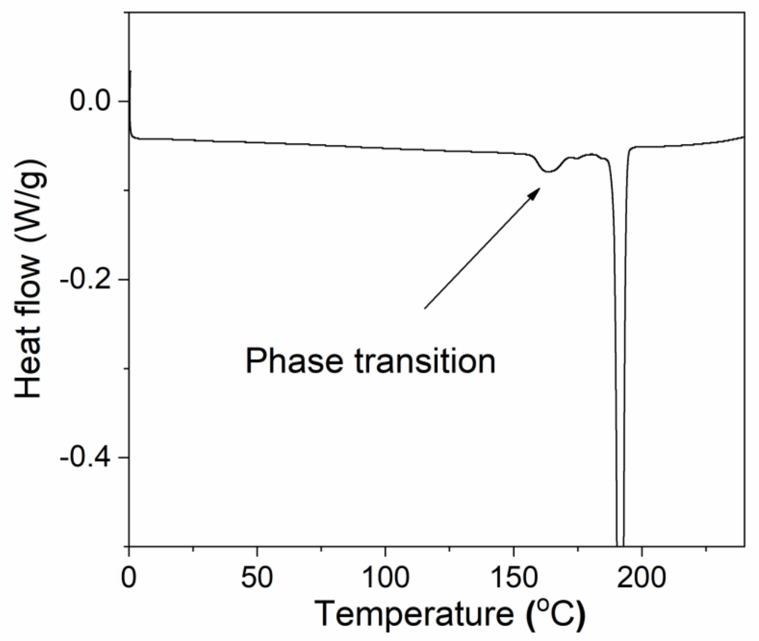

Figure S1. Details of the minor peak below 170 °C in the DSC trace of [$Cu_4I_4(PPh_2Et)_4$] crystal.

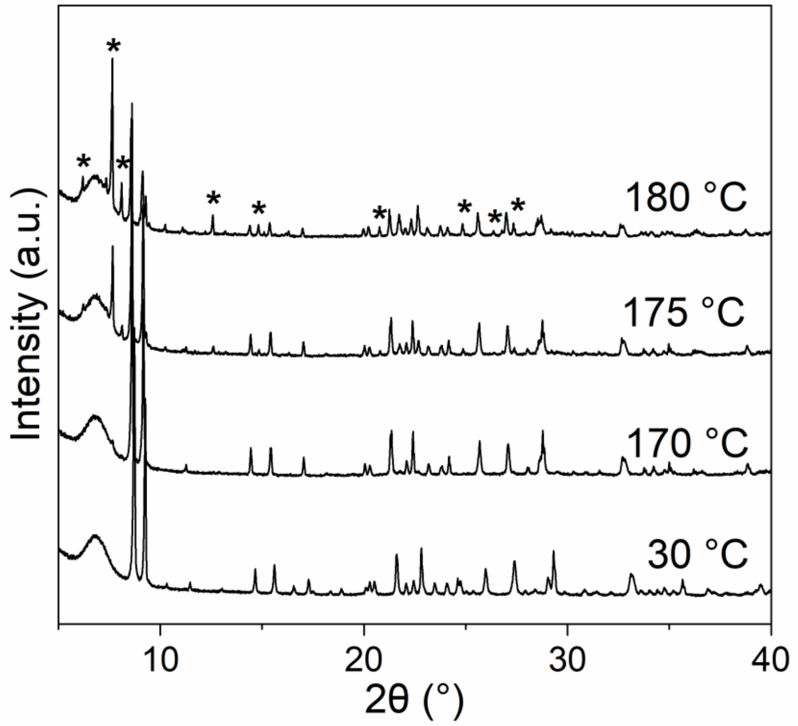

Figure S2. Temperature-dependent powder X-ray diffraction (XRD) patterns of [$Cu_4I_4(PPh_2Et)_4$] crystal. The peaks indicated by asterisk (*) appear at high temperature, which demonstrates the phase transition of the nanocluster.

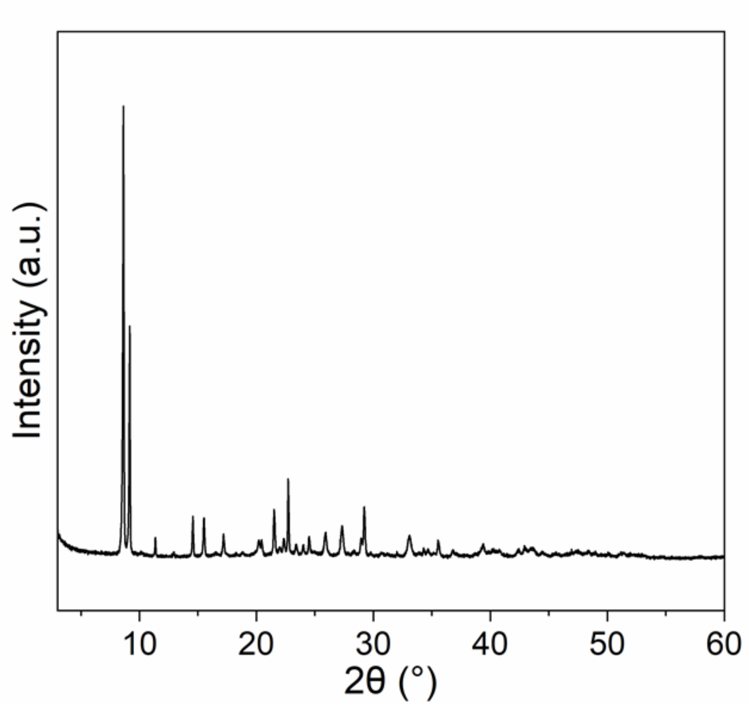

Figure S3. XRD pattern of $[Cu_4I_4(PPh_2Et)_4]$ prepared by slow cooling from the melt.

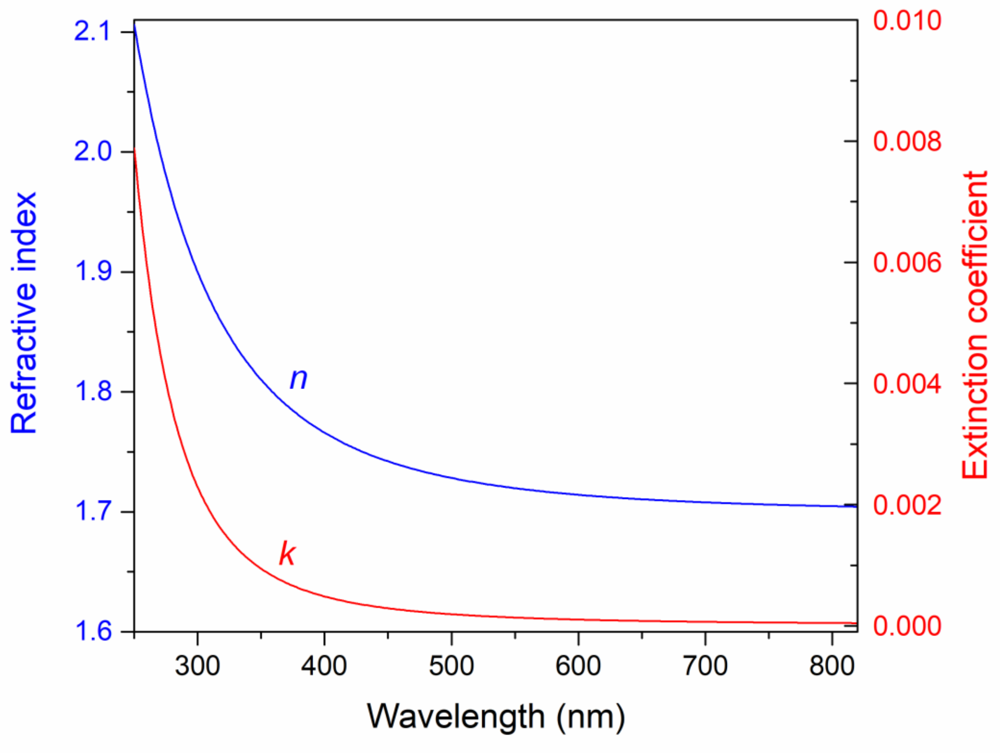

Figure S4. Refractive index (*n*) and extinction coefficient (*k*) of $[Cu_4I_4(PPh_2Et)_4]$ glass.

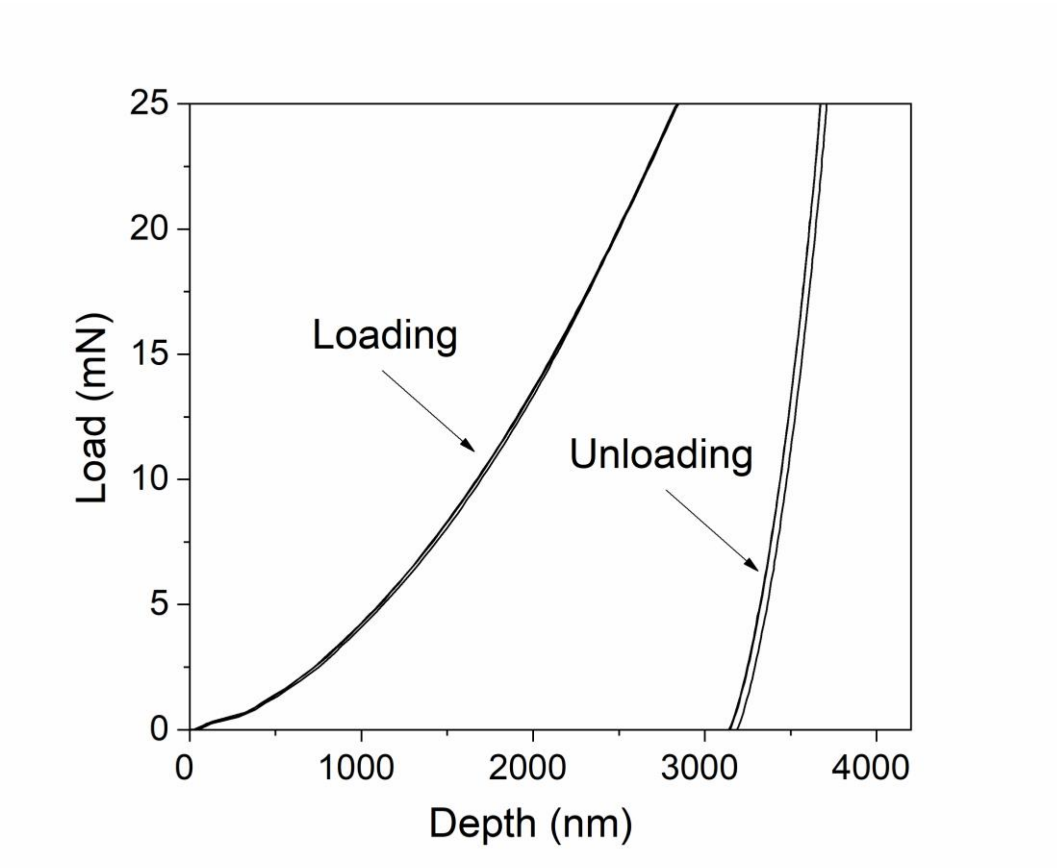

Figure S5. Load-displacement curves obtained in the nanoindentation experiment of bulk [$Cu_4I_4(PPh_2Et)_4$] glass at room temperature (23 ℃).

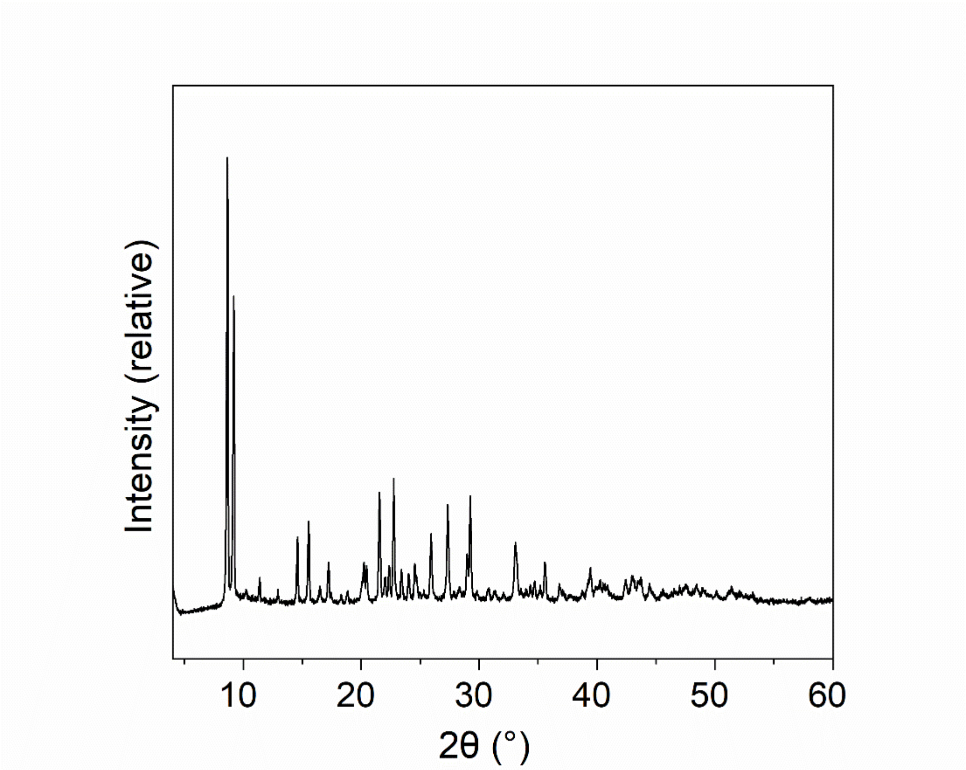

Figure S6. XRD pattern of [$Cu_4I_4(PPh_2Et)_4$] prepared by heating the glass at 100 ^{o}C for 1 h.

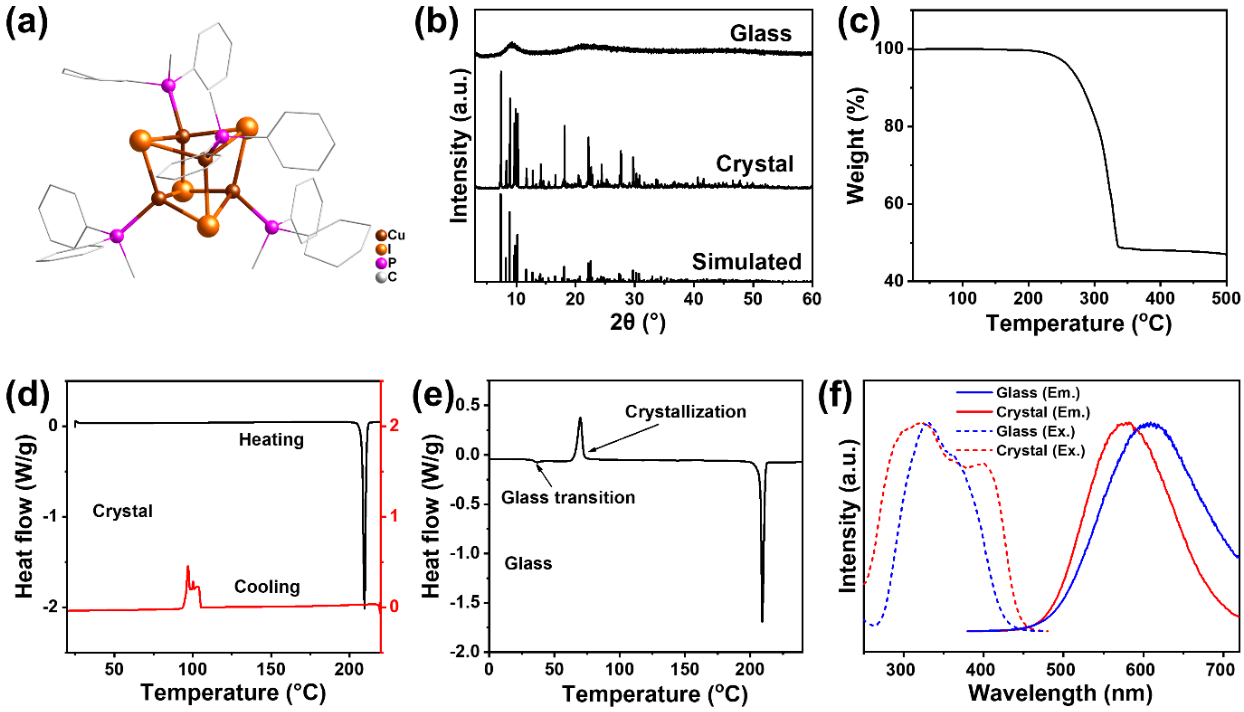

Figure S7. Characterizations of [$Cu_4I_4(PPh_2Me)_4$] crystal and glass. (a) Crystal structure of [$Cu_4I_4(PPh_2Me)_4$] nanocluster. (b) XRD patterns of [$Cu_4I_4(PPh_2Me)_4$] crystal and glass. (c) Thermogravimetric analysis of [$Cu_4I_4(PPh_2Me)_4$] crystal. Differential scanning calorimetry (DSC) traces for [$Cu_4I_4(PPh_2Me)_4$] (d) crystal and (e) glass. (f) Excitation and emission spectra of [$Cu_4I_4(PPh_2Me)_4$] crystal and glass.

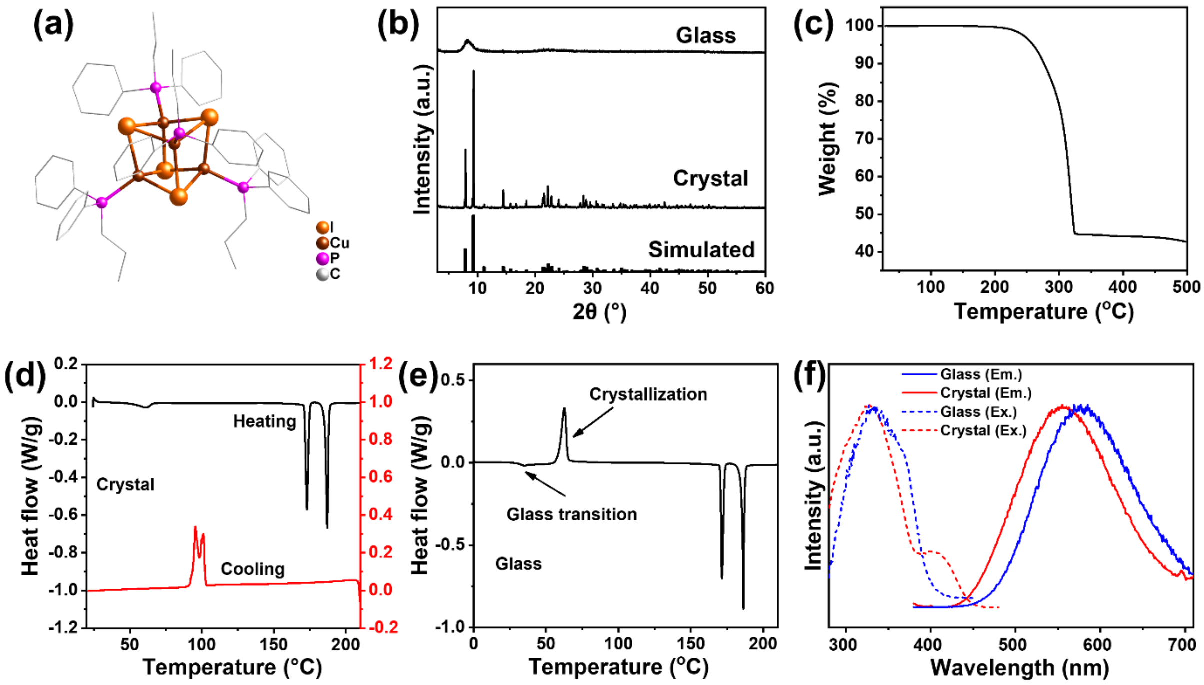

Figure S8. Characterizations of [$Cu_4I_4(PPh_2Pr)_4$] crystal and glass. (a) Crystal structure of [$Cu_4I_4(PPh_2Pr)_4$] nanocluster. (b) XRD patterns of [$Cu_4I_4(PPh_2Pr)_4$] crystal and glass. (c) Thermogravimetric analysis of [$Cu_4I_4(PPh_2Pr)_4$] crystal. DSC traces for [$Cu_4I_4(PPh_2Pr)_4$] (d) crystal and (e) glass. (f) Excitation and emission spectra of [$Cu_4I_4(PPh_2Pr)_4$] crystal and glass.

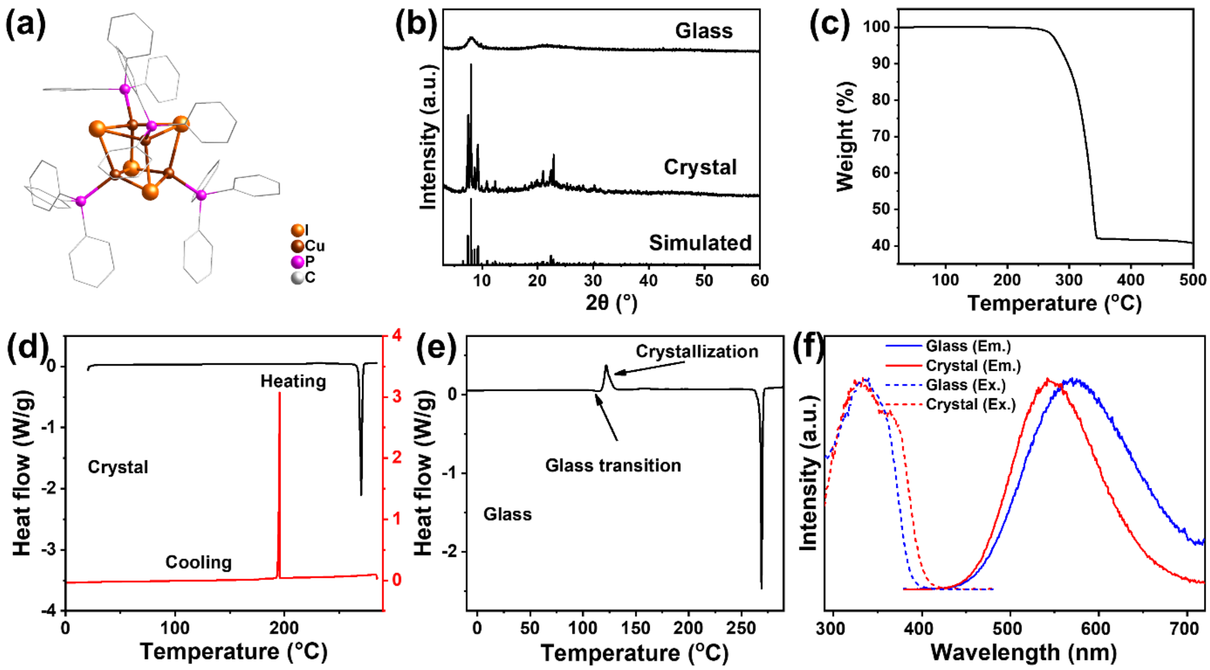

Figure S9. Characterizations of [$Cu_4I_4(PPh_3)_4$] crystal and glass. (a) Crystal structure of [$Cu_4I_4(PPh_3)_4$] nanocluster. (b) XRD patterns of [$Cu_4I_4(PPh_3)_4$] crystal and glass. (c) Thermogravimetric analysis of [$Cu_4I_4(PPh_3)_4$] crystal. DSC traces for [$Cu_4I_4(PPh_3)_4$] (d) crystal and (e) glass. (f) Excitation and emission spectra of [$Cu_4I_4(PPh_3)_4$] crystal and glass.

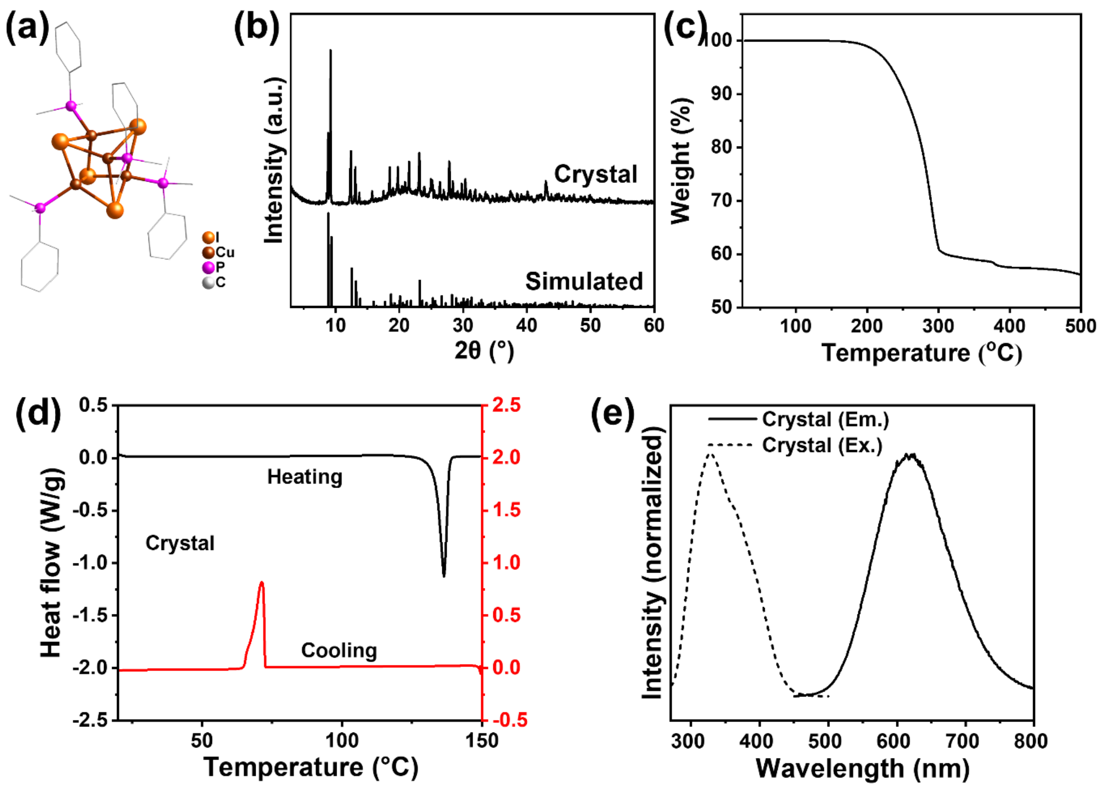

Figure S10. Characterizations of [$Cu_4I_4(PPhMe_2)_4$] crystal. (a) Crystal structure, (b) XRD pattern, (c) thermogravimetric analysis, (d) DSC traces, and (e) Excitation and emission spectra of [$Cu_4I_4(PPhMe_2)_4$] nanocluster.

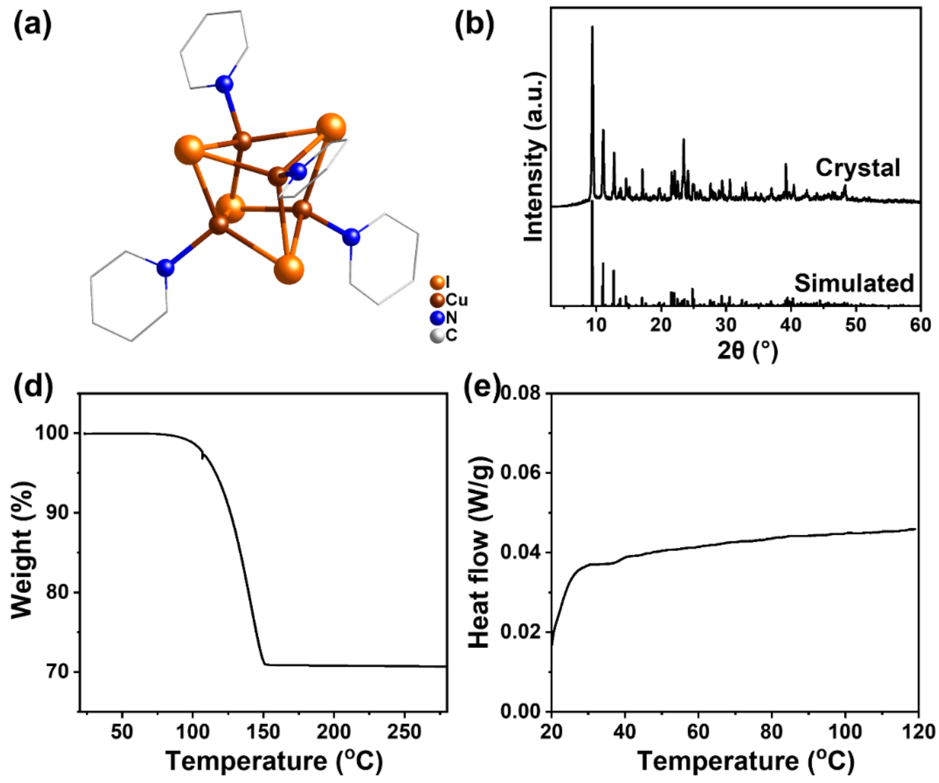

Figure S11. Characterizations of [$Cu_4I_4Py_4$] nanocluster. (a) Crystal structure, (b) XRD pattern, (c) thermogravimetric (TG) analysis, and (d) DSC trace of [$Cu_4I_4Py_4$] nanocluster.

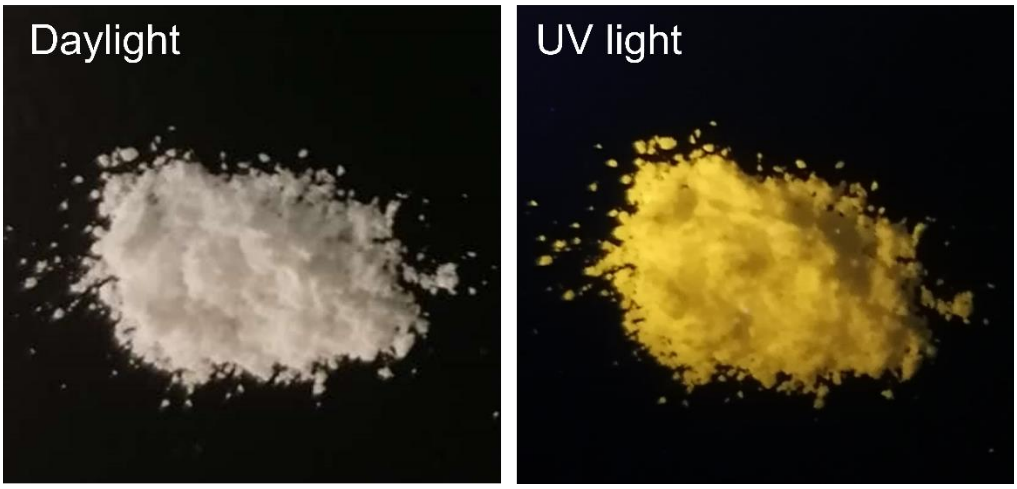

Figure S12. Photographs of [$Cu_4I_4(PPh_2Et)_4$] crystal under daylight (left) and UV light (right).

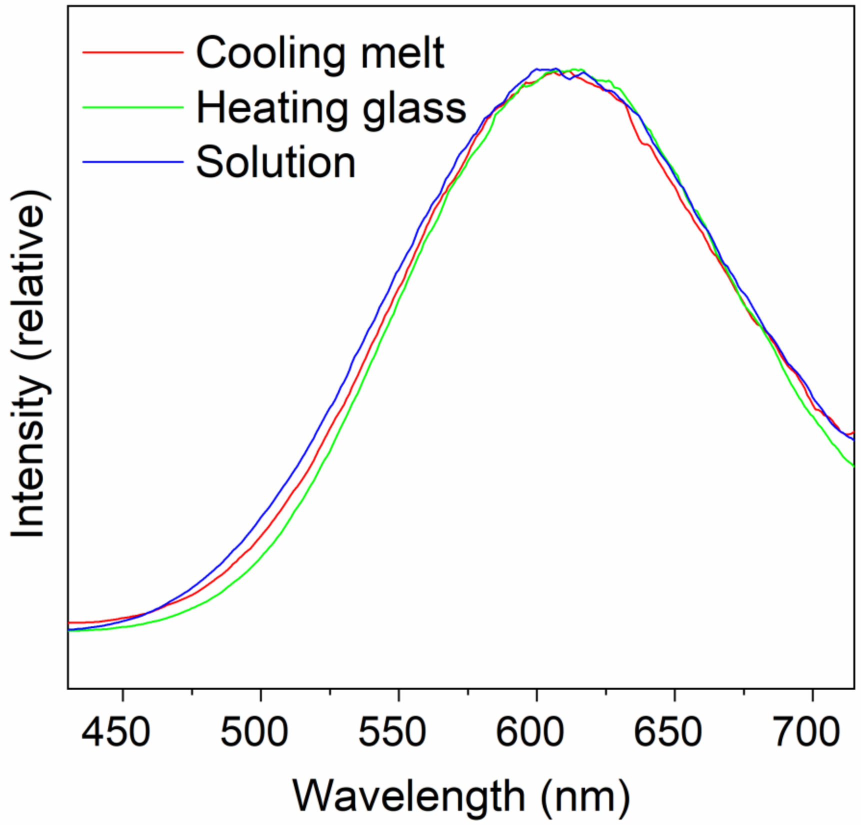

Figure S13. Photoluminescence spectra of [$Cu_4I_4(PPh_2Et)_4$] crystals prepared by slow cooing the melt, heating the glass, and anti-solvent diffusion (solution).

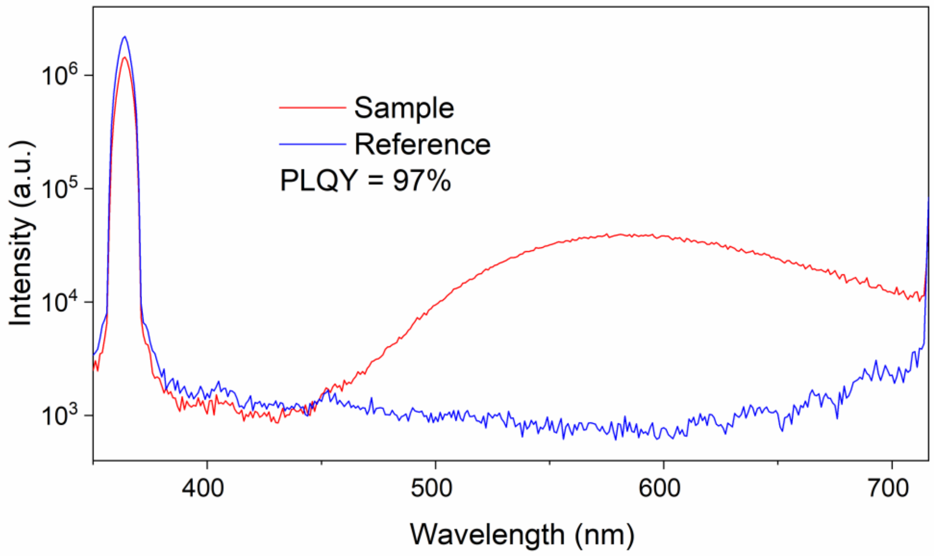

Figure S14. Photoluminescence quantum yield (PLQY) data of $[Cu_4I_4(PPh_2Et)_4]$ glass.

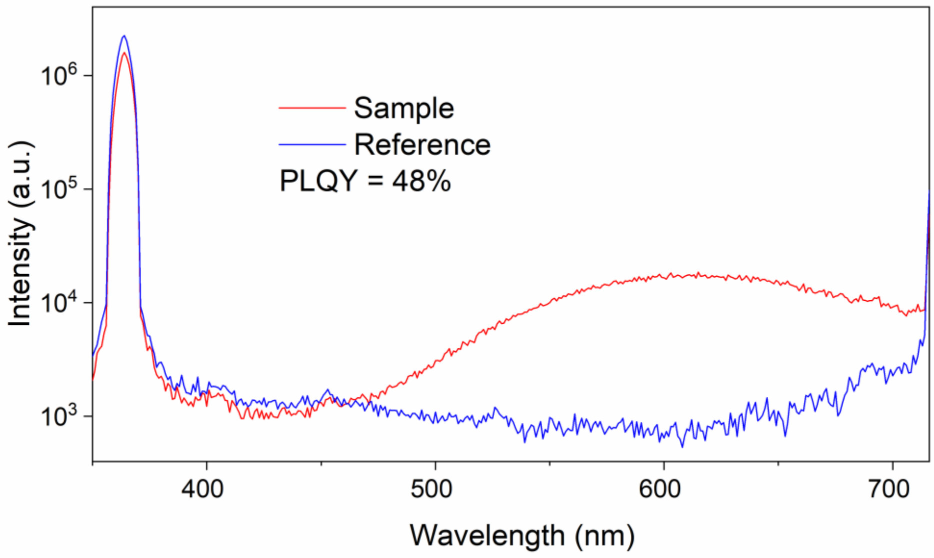

Figure S15. Photoluminescence quantum yield (PLQY) data of $[Cu_4I_4(PPh_2Et)_4]$ crystal.

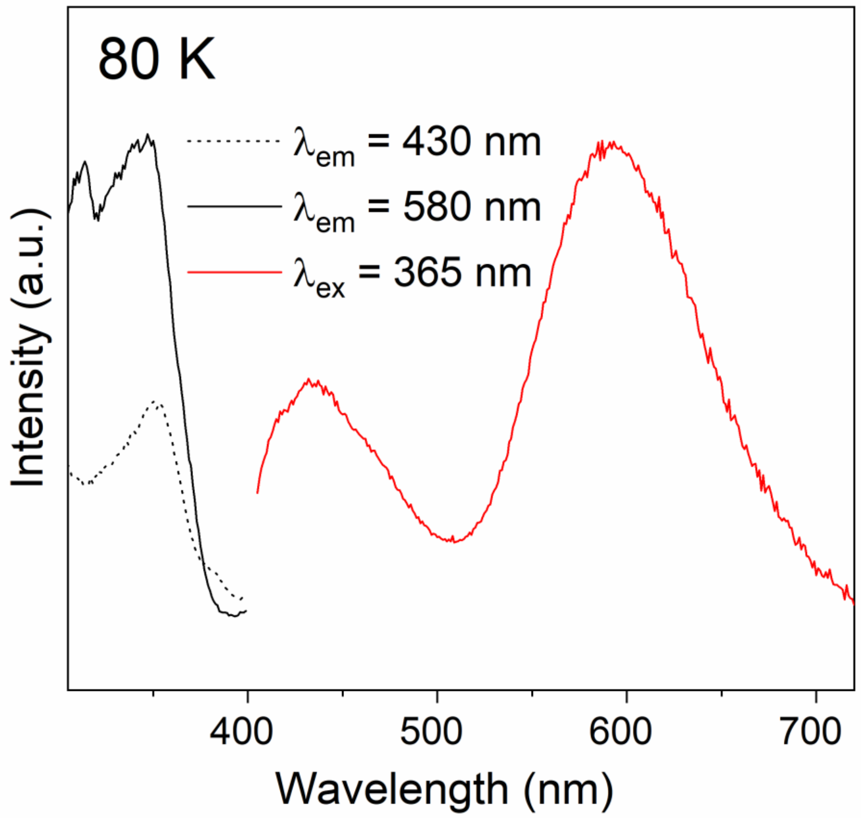

Figure S16. Excitation and emission spectra of [$Cu_4I_4(PPh_2Et)_4$] glass at 80 K.

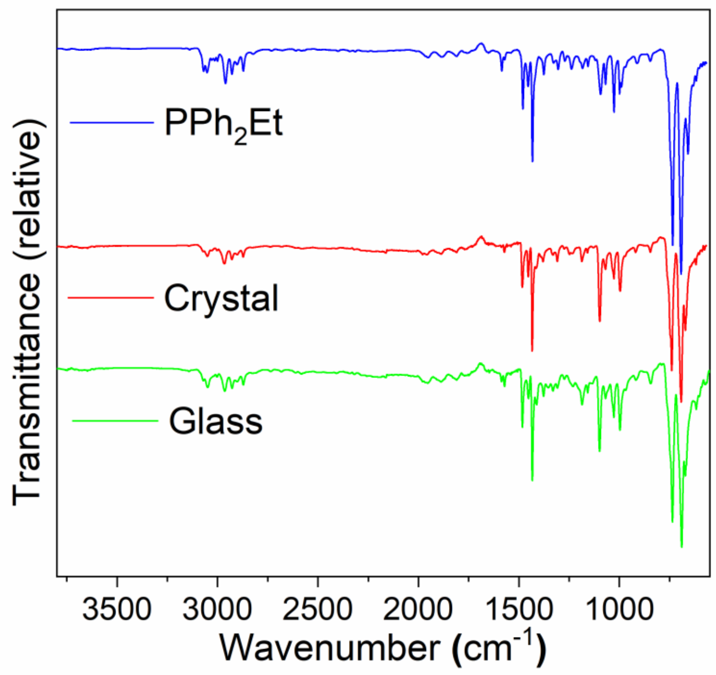

Figure S17. Fourier transform infrared (FTIR) spectra of [$Cu_4I_4(PPh_2Et)_4$] glass, crystal, and PPh_2Et.

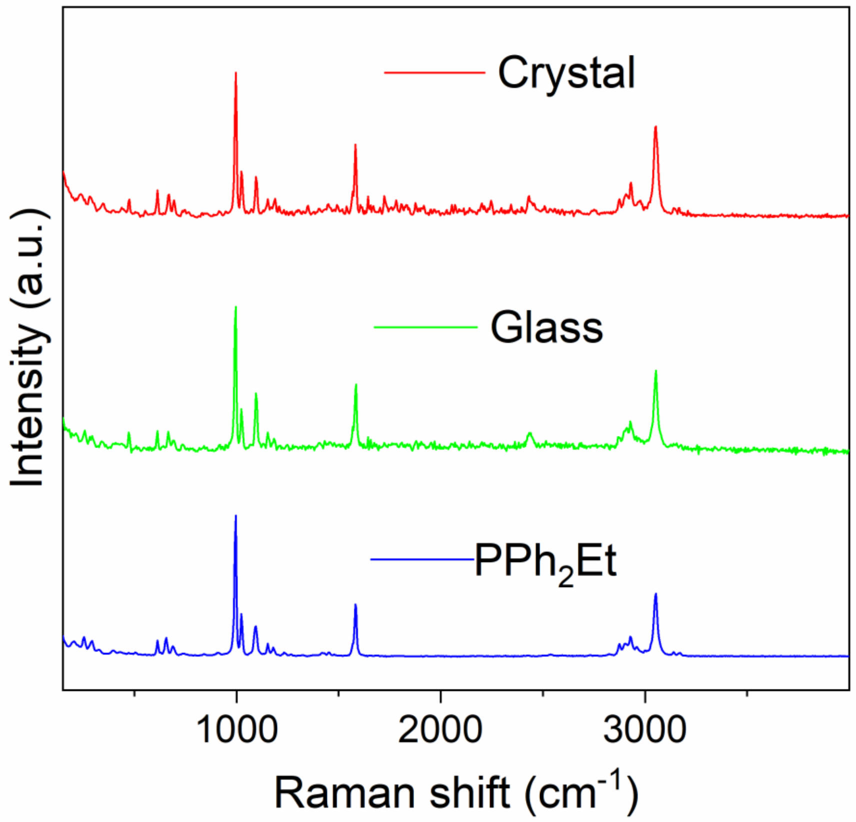

Figure S18. Raman spectra of $[Cu_4I_4(PPh_2Et)_4]$ glass and crystal.

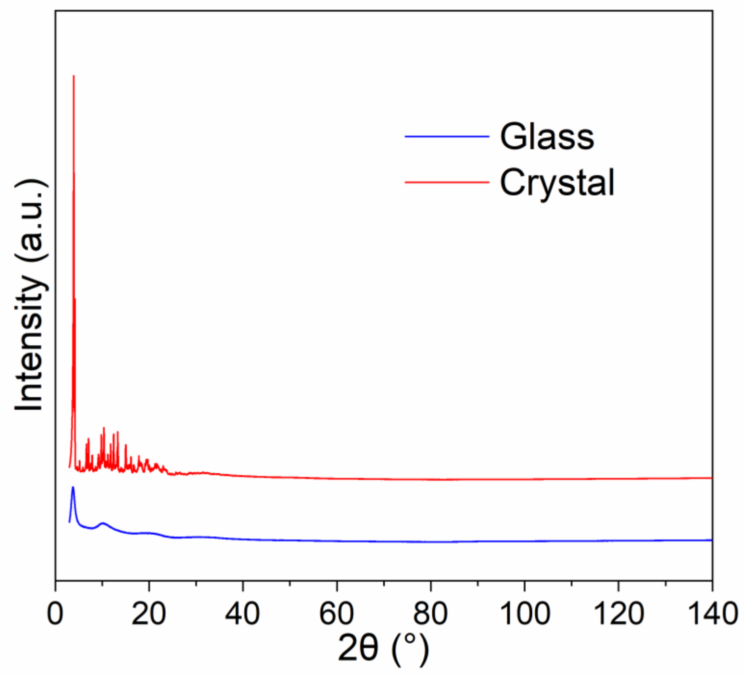

Figure S19. The X-ray total scattering data of [$Cu_4I_4(PPh_2Et)_4$] glass and crystal. The data were collected using a molybdenum target (Mo Kα, λ = 0.71073 Å).

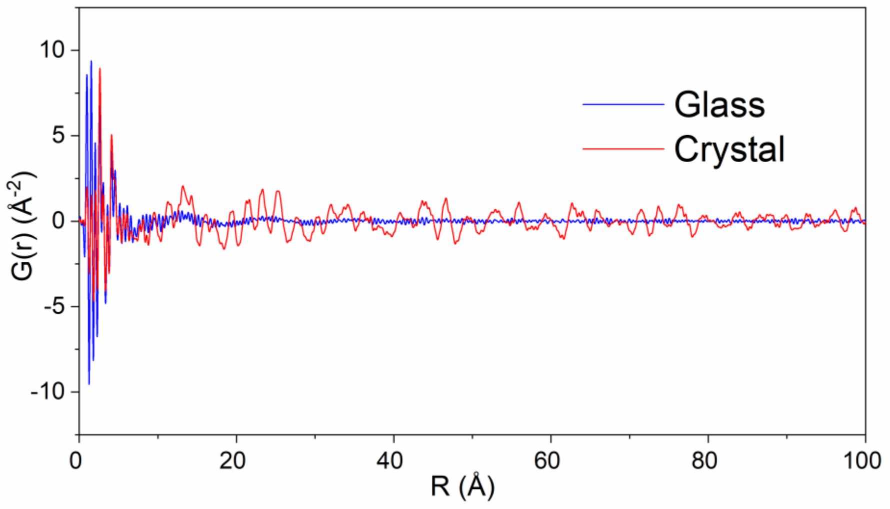

Figure S20. Global pair distribution functions (PDFs) of [$Cu_4I_4(PPh_2Et)_4$] crystal and glass.

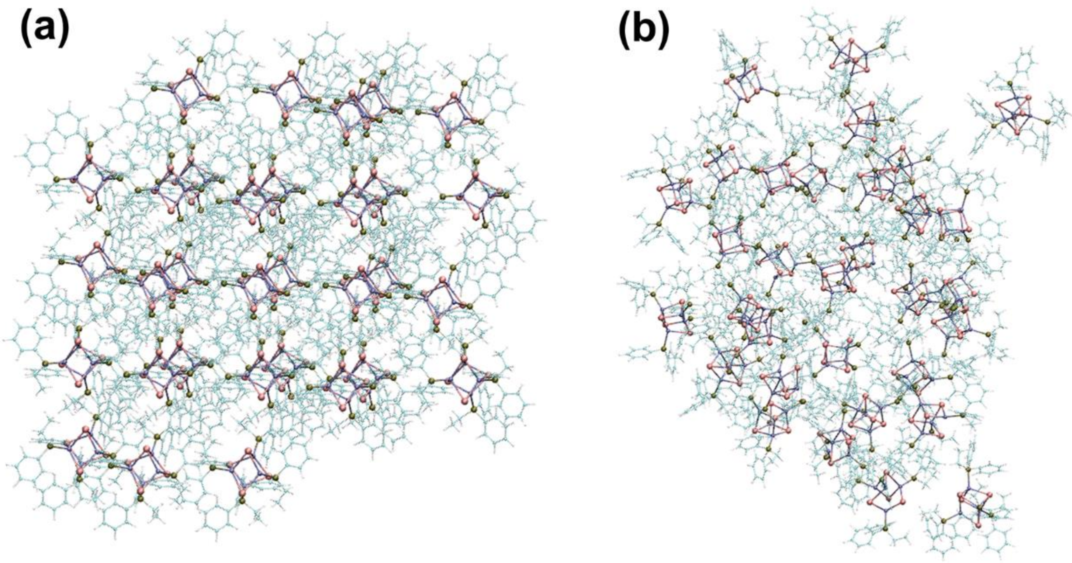

Figure S21. (a) Optimized structure of $[Cu_4I_4(PPh_2Et)_4]$ crystal and (b) AIMD structure of $[Cu_4I_4(PPh_2Et)_4]$ glass derived from a 2x2x2 supercell.

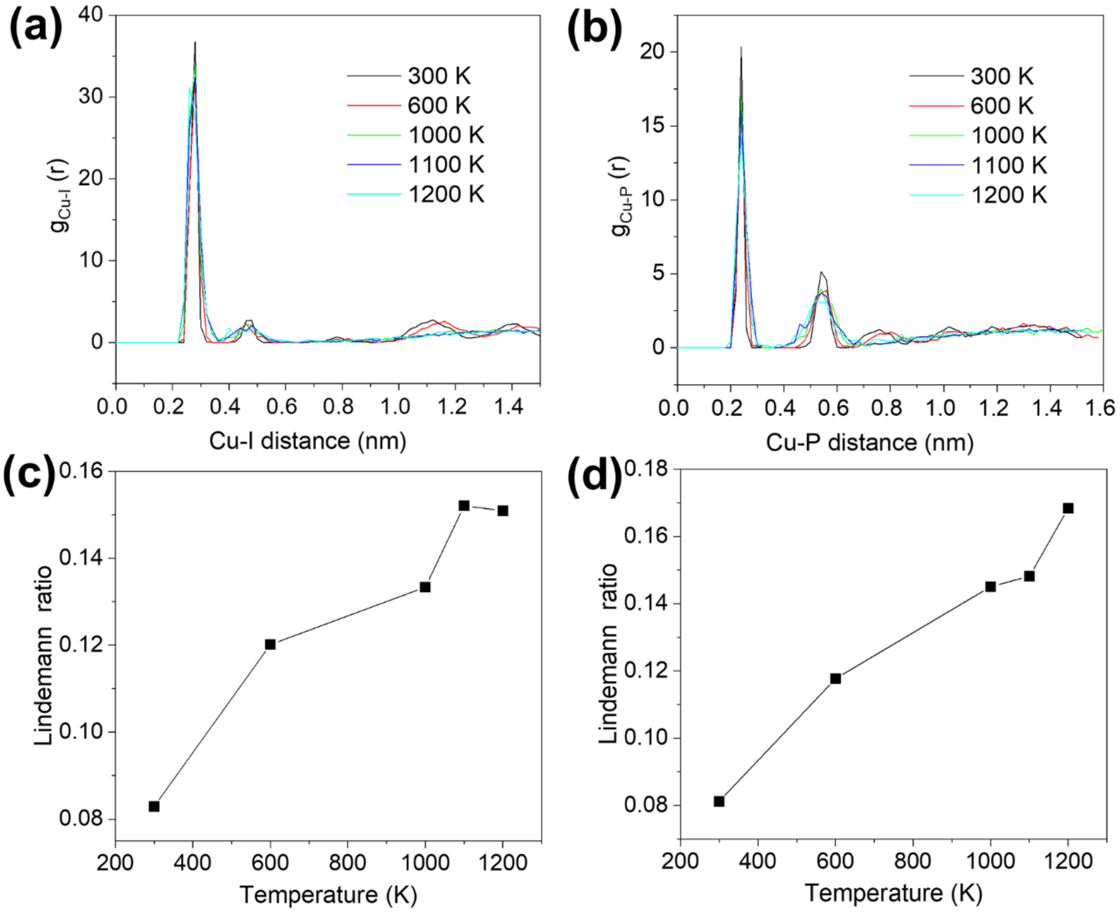

Figure S22. Evolution of the partial radial distribution function $g_{ij}(r)$ for (a) Cu–I distance and (b) Cu-P distance at varied temperatures derived from a 2x2x2 supercell AIMD simulation. The generalized Lindemann ratio at varied temperatures for (c) Cu–I bond and (d) Cu–P bond derived from a 2x2x2 supercell.

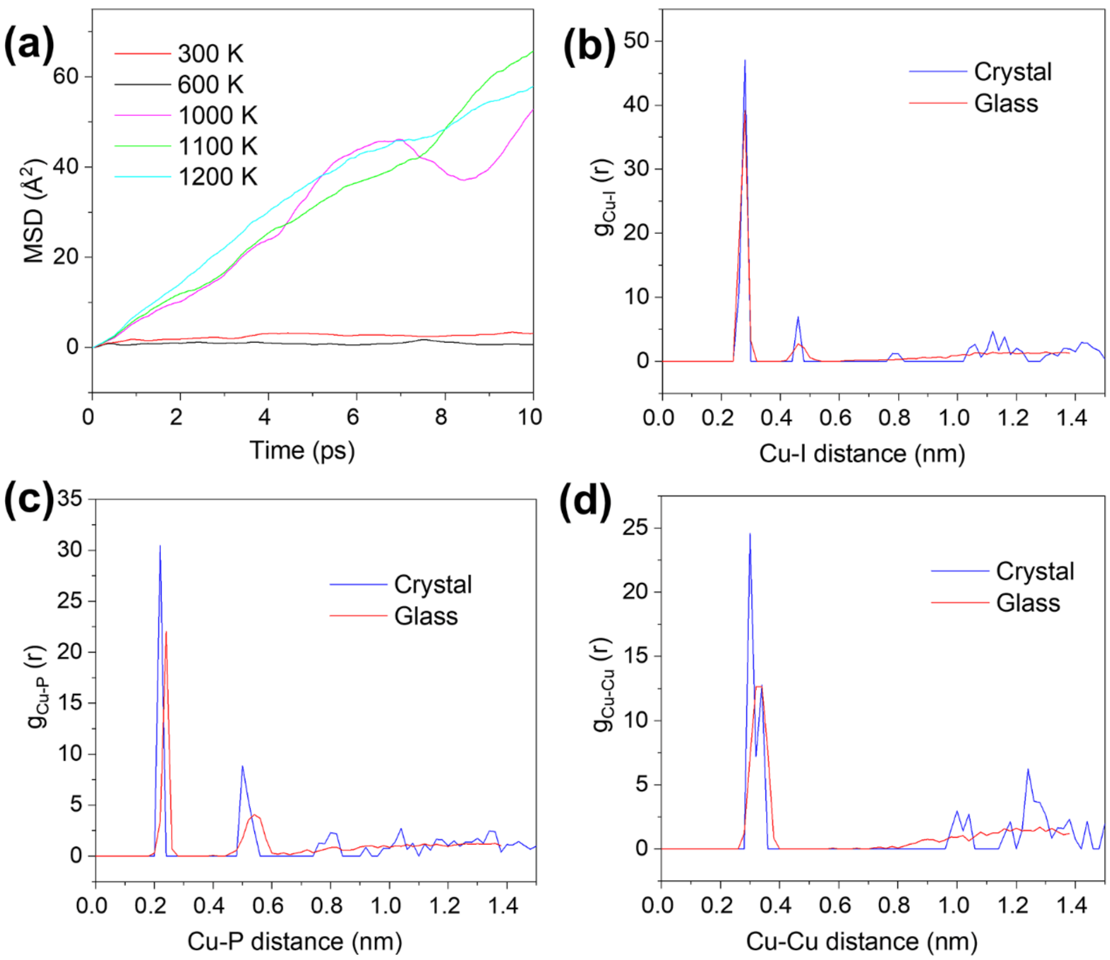

Figure S23. (a) Mean square displacement (MSD) in [$Cu_4I_4(PPh_2Et)_4$] at varied temperatures based on a 2x2x2 supercell AIMD simulation. The partial radial distribution function $g_{ij}(r)$ for (b) Cu–I distance, (c) Cu–P distance, and (d) Cu–Cu distance in optimized [$Cu_4I_4(PPh_2Et)_4$] crystal and AIMD [$Cu_4I_4(PPh_2Et)_4$] glass derived from a 2x2x2 supercell.

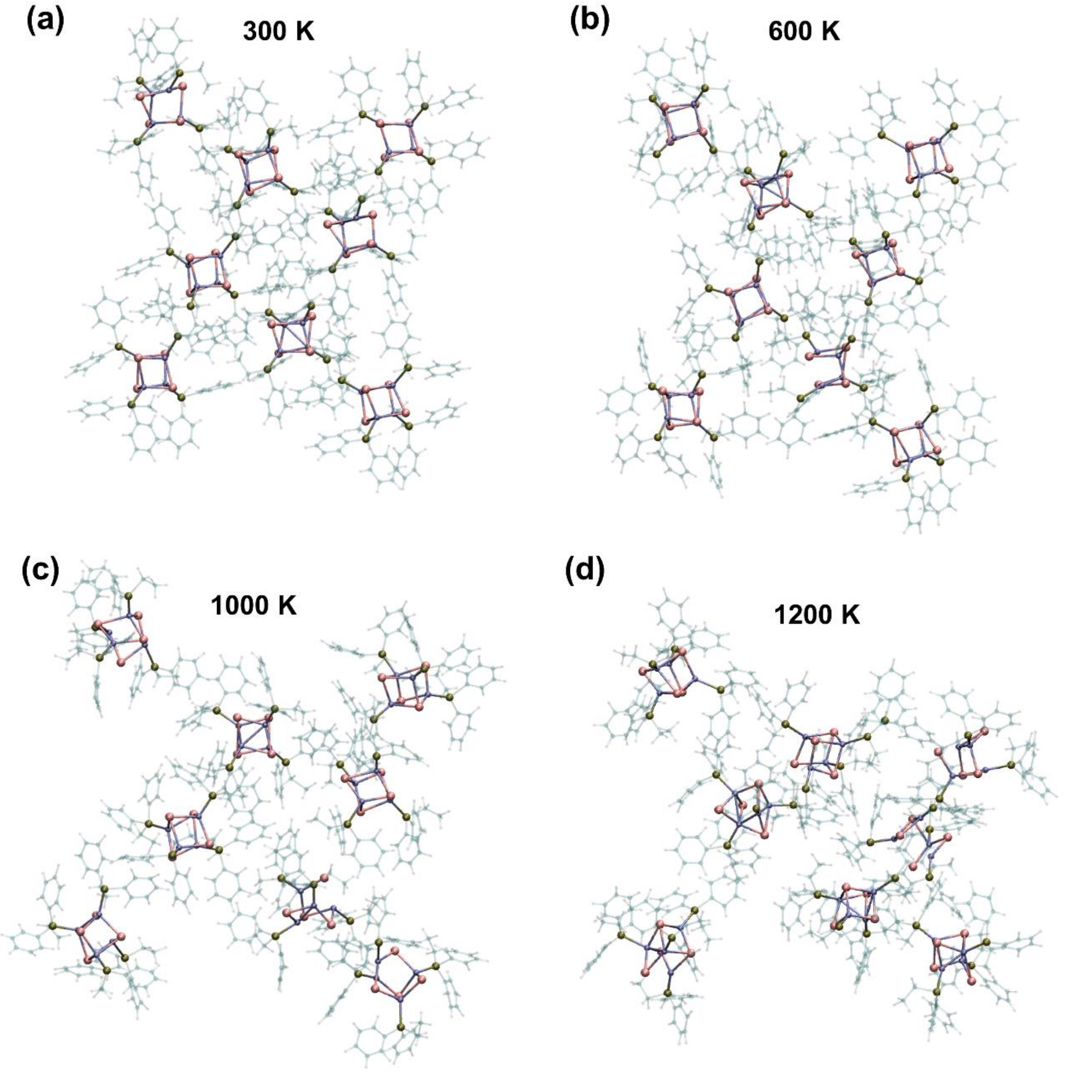

Figure S24. Screenshots of AIMD simulation of $[Cu_4I_4(PPh_2Et)_4]$ crystal at various temperatures.

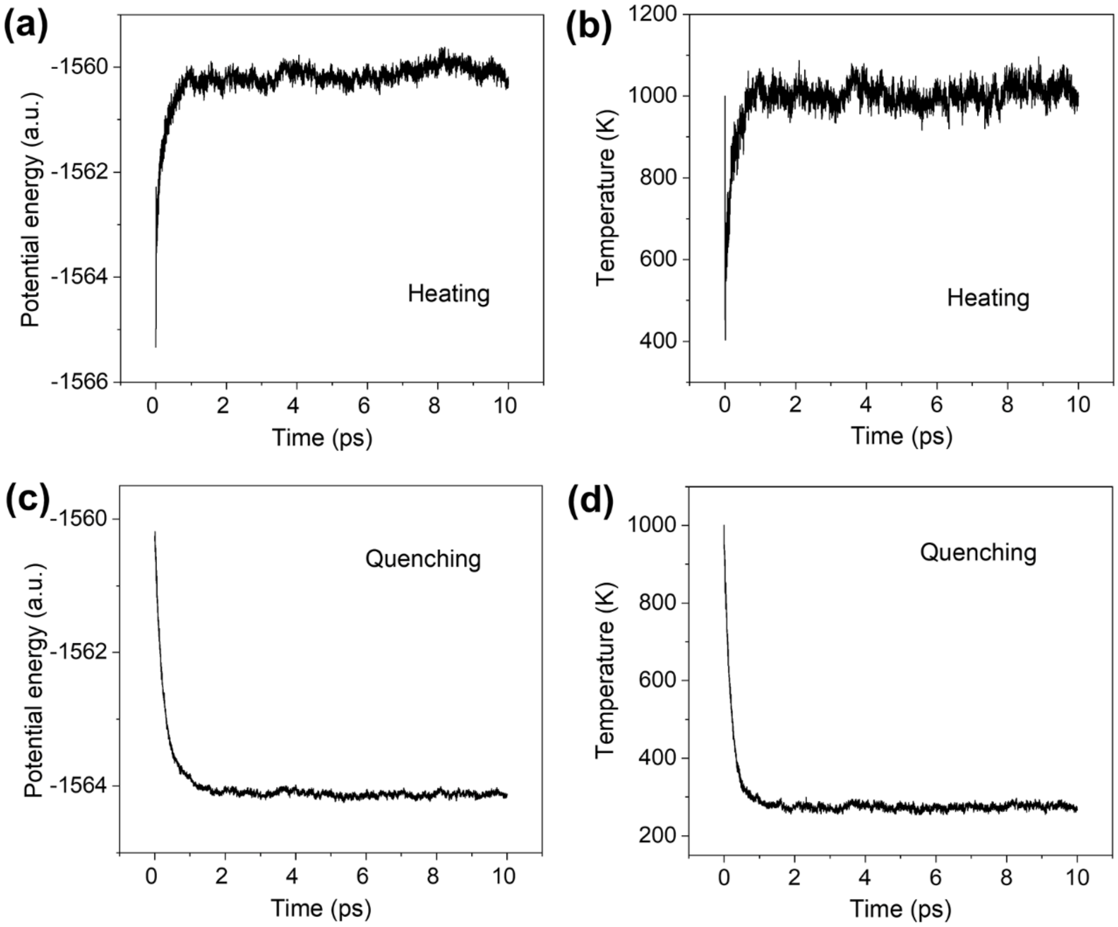

Figure S25. The temperature and potential energy vs. time plots throughout the whole simulation derived from a 1x1x2 supercell. (a, c) The potential energy vs. time plots and (b, d) temperature vs. time plots for the (a, b) heating and (c, d) quenching process derived from a 1x1x2 supercell.

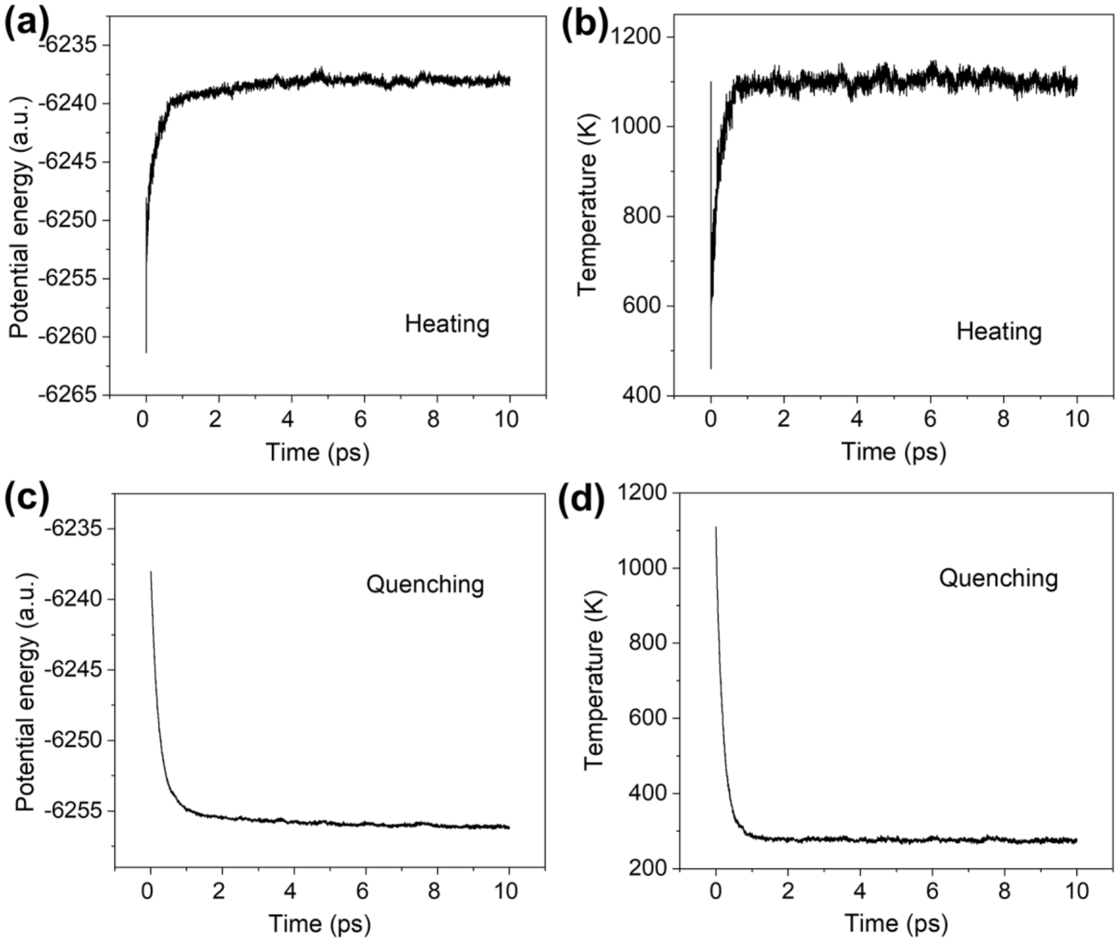

Figure S26. The temperature and potential energy vs. time plots throughout the whole simulation derived from a 2x2x2 supercell. (a, c) The potential energy vs. time plots and (b, d) temperature vs. time plots for the (a, b) heating and (c, d) quenching process derived from a 2x2x2 supercell.

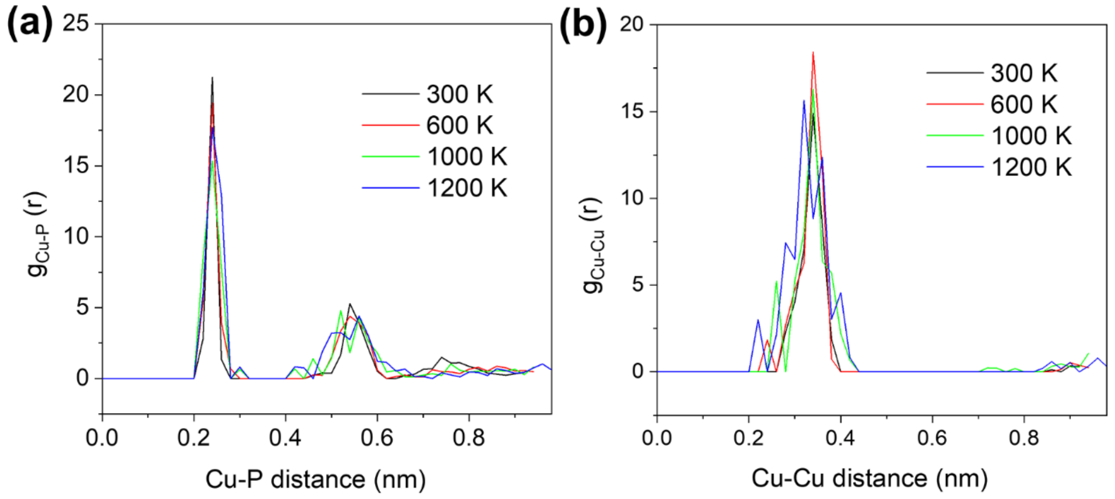

Figure S27. Evolution of the partial radial distribution function $g_{ij}(r)$ for (a) Cu–P and (b) Cu–Cu distance in $[Cu_4I_4(PPh_2Et)_4]$ at varied temperatures from 300 K to 1200 K based on AIMD.

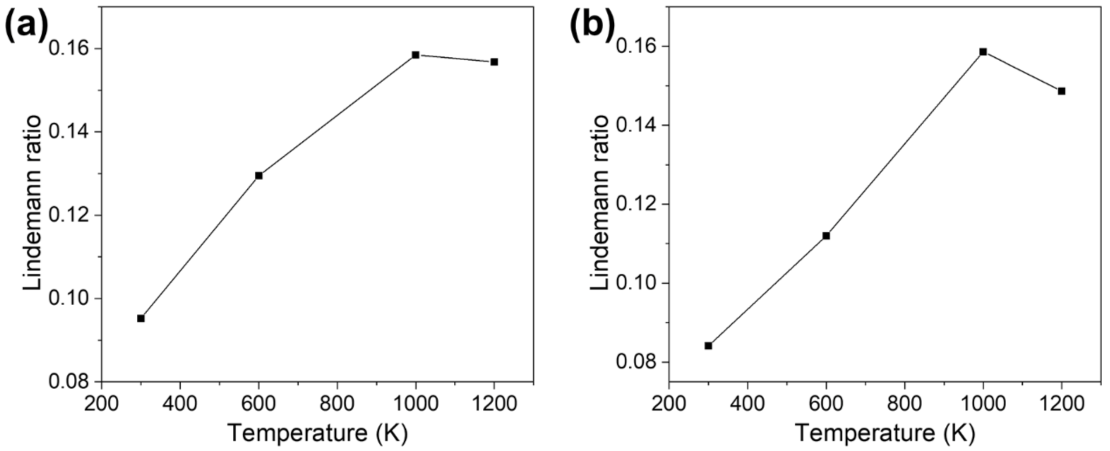

Figure S28. The generalized Lindemann ratio at varied temperatures for (a) Cu–P bond and (b) Cu–I bond.

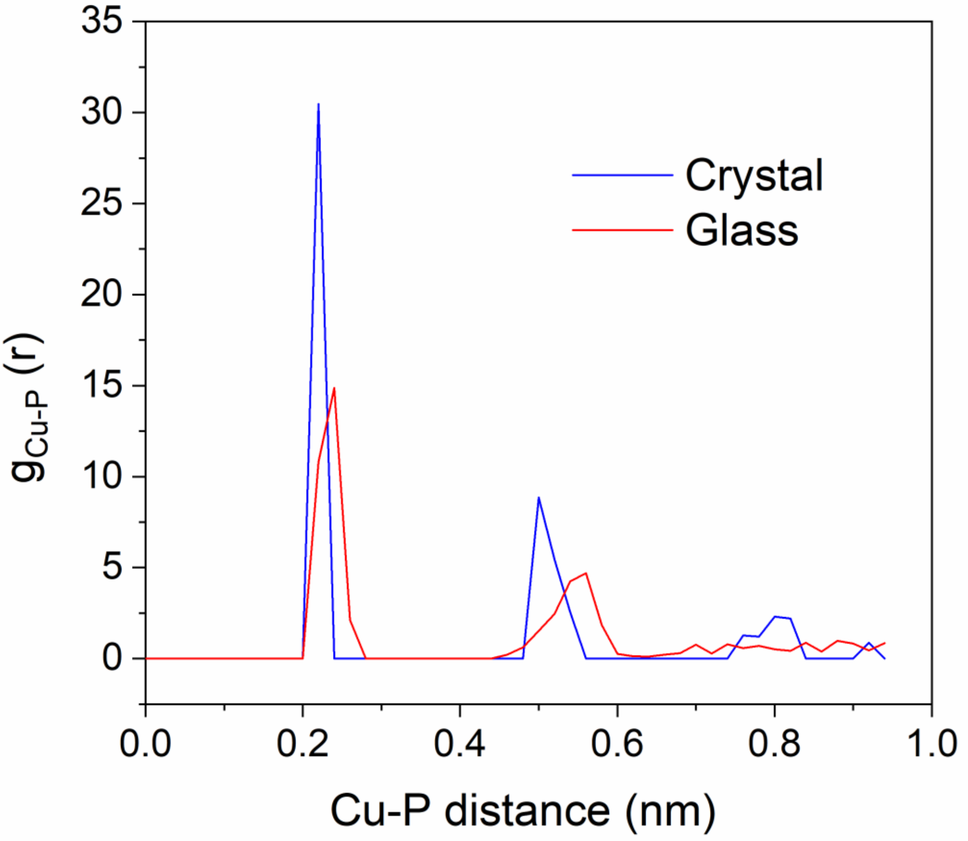

Figure S29. The partial radial distribution function $g_{ij}(r)$ for Cu–P distance in optimized [$Cu_4I_4(PPh_2Et)_4$] crystal and AIMD [$Cu_4I_4(PPh_2Et)_4$] glass.

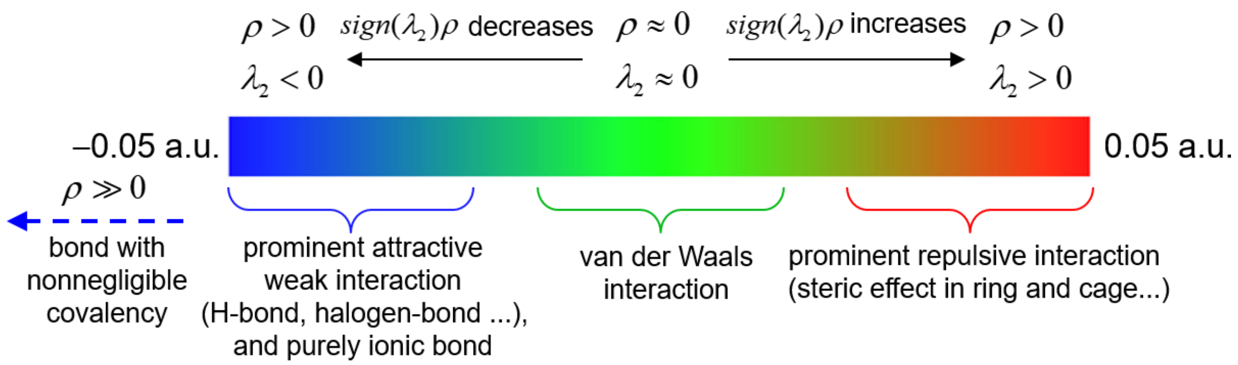

Figure S30. Color-bar of independent gradient model based on Hirshfeld partition (IGMH) analysis.

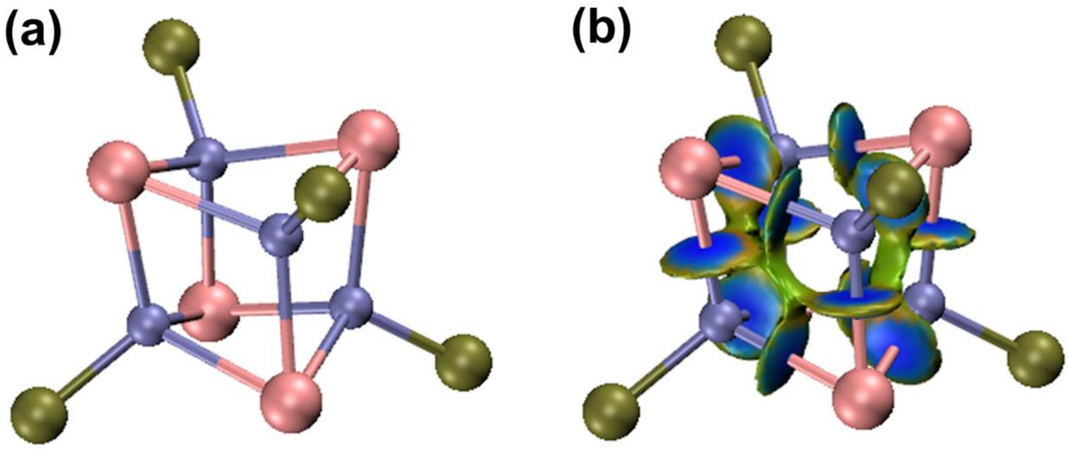

Figure S31. The weak interactions withing the $[Cu_4I_4P_4]$ core of $[Cu_4I_4(PPh_2Et)_4]$ crystal analyzed by IGMH. Purple: Cu; tan; P; and pink: I. All carbon and hydrogen atoms are omitted.

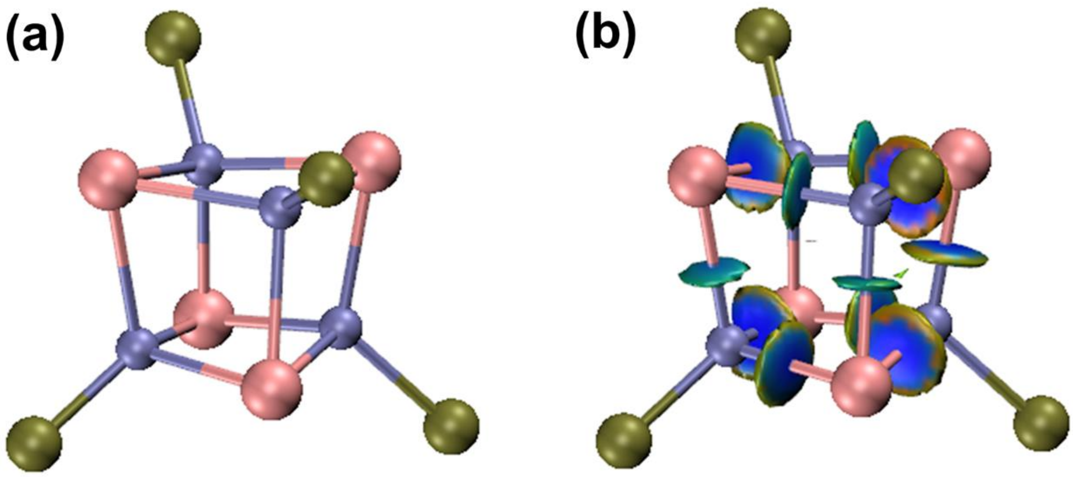

Figure S32. The weak interactions withing the $[Cu_4I_4P_4]$ core of $[Cu_4I_4(PPh_2Et)_4]$ glass analyzed by IGMH. Purple: Cu; tan; P; and pink: I. All carbon and hydrogen atoms are omitted.

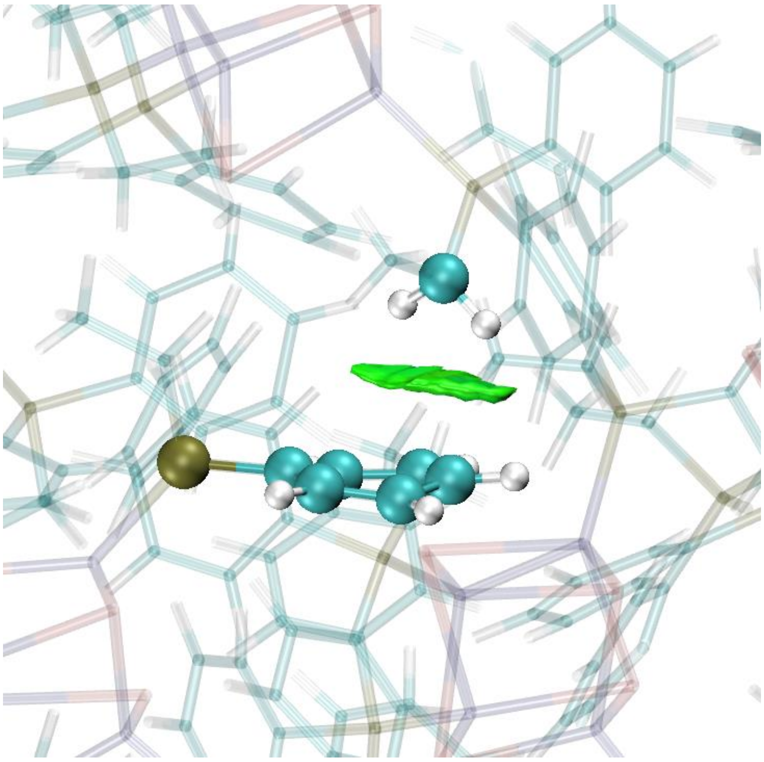

Figure S33. The weak interactions analyzed by IGMH for -CH_2- to benzene ring in AIMD $[Cu_4I_4(PPh_2Et)_4]$ glass.

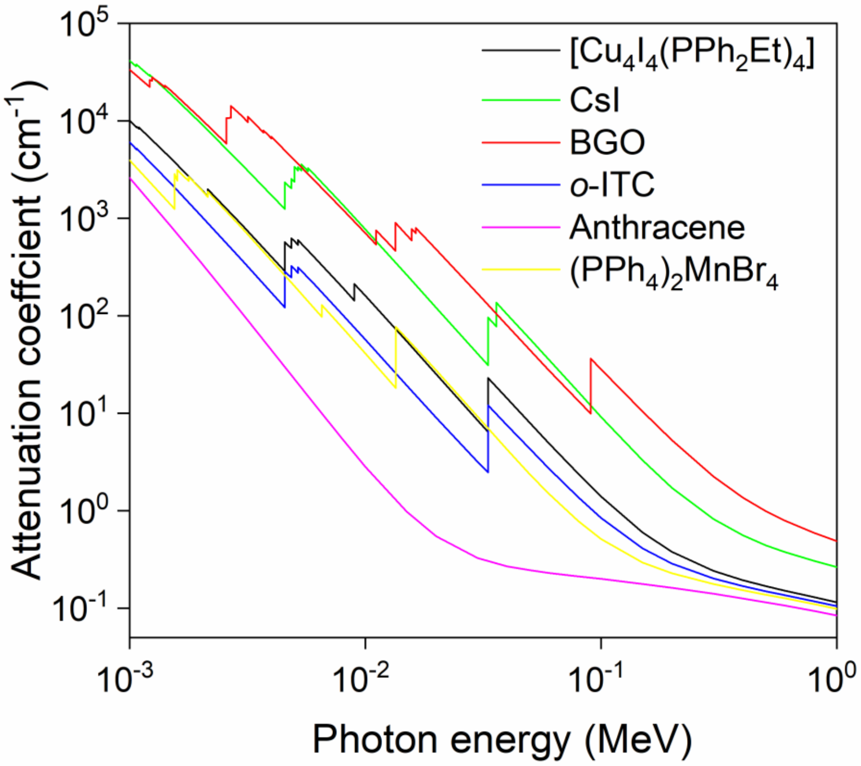

Figure S34. X-ray attenuation coefficient as a function of photon energy for $[Cu_4I_4(PPh_2Et)_4]$, CsI, BGO, *o*-ITC, anthracene, and $(PPh_4)_2MnBr_4$.

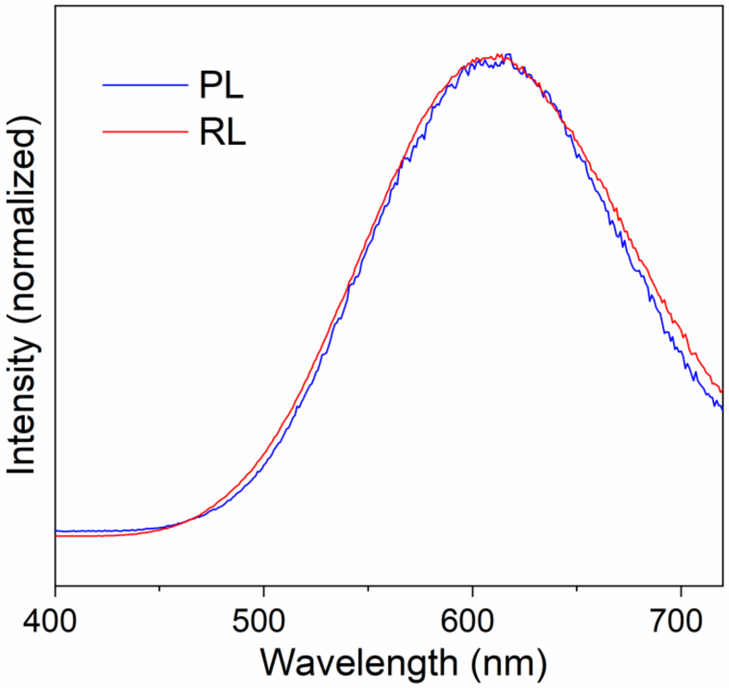

Figure S35. Comparison of the PL and RL spectra for [$Cu_4I_4(PPh_2Et)_4$] crystal.

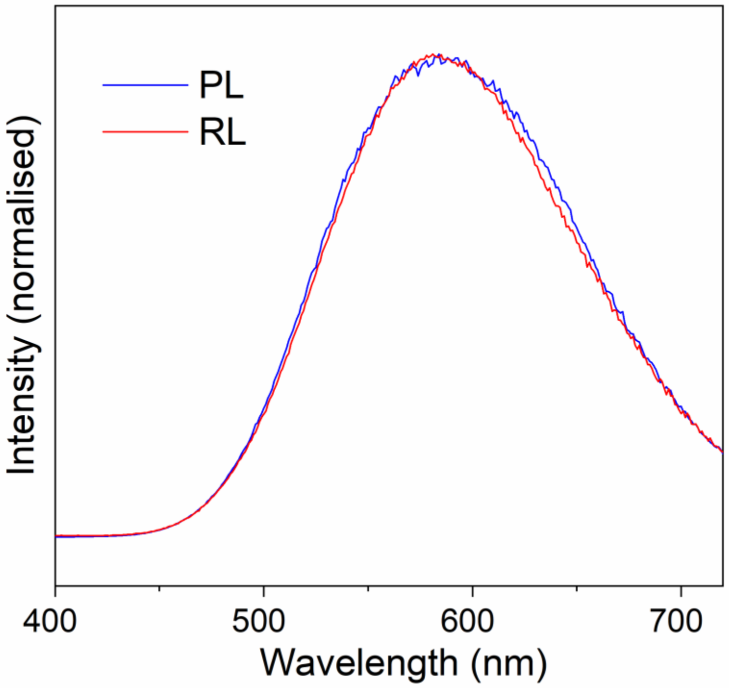

Figure S36. Comparison of the PL and RL spectra for [$Cu_4I_4(PPh_2Et)_4$] glass.

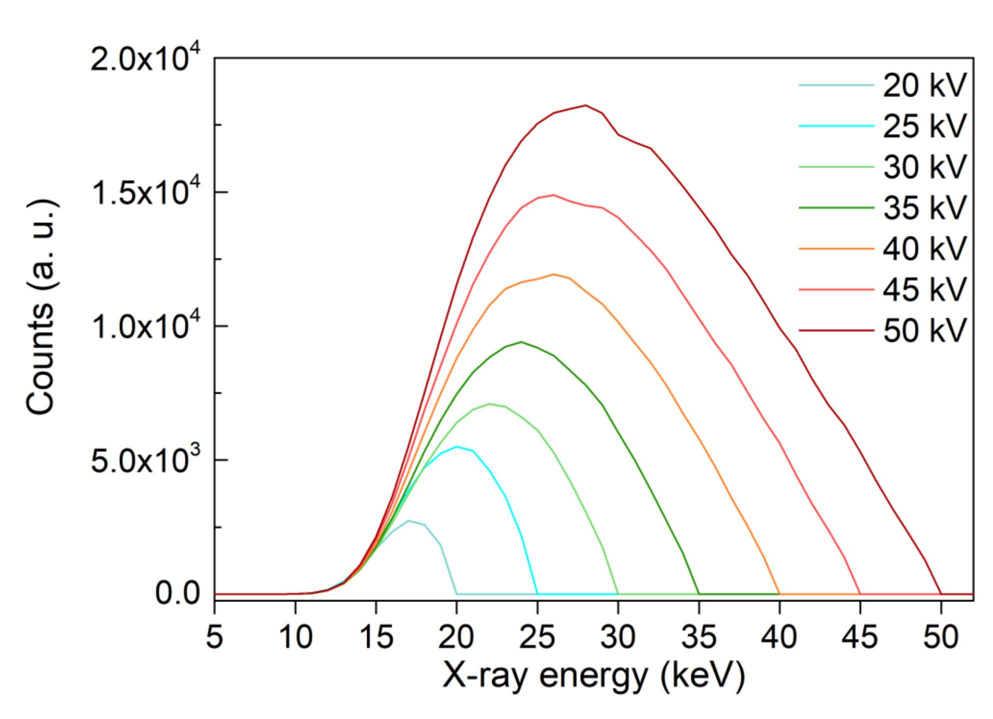

Figure S37. The energy spectrum of the X-ray source up to 50 keV.

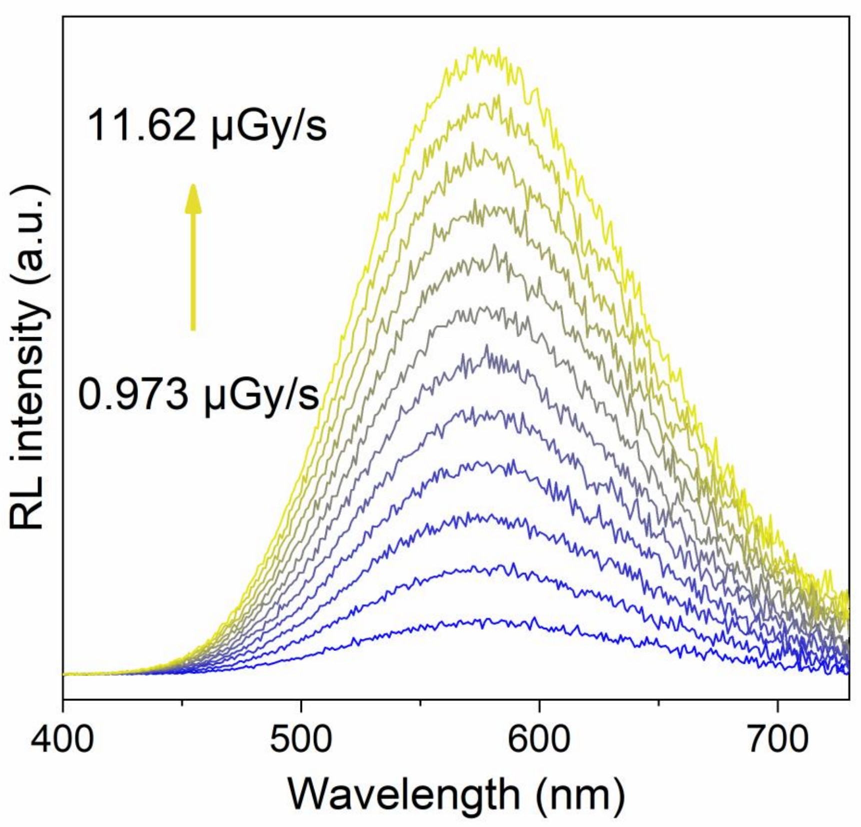

Figure S38. RL spectra of $[Cu_4I_4(PPh_2Et)_4]$ glass under varied X-ray dose rates.

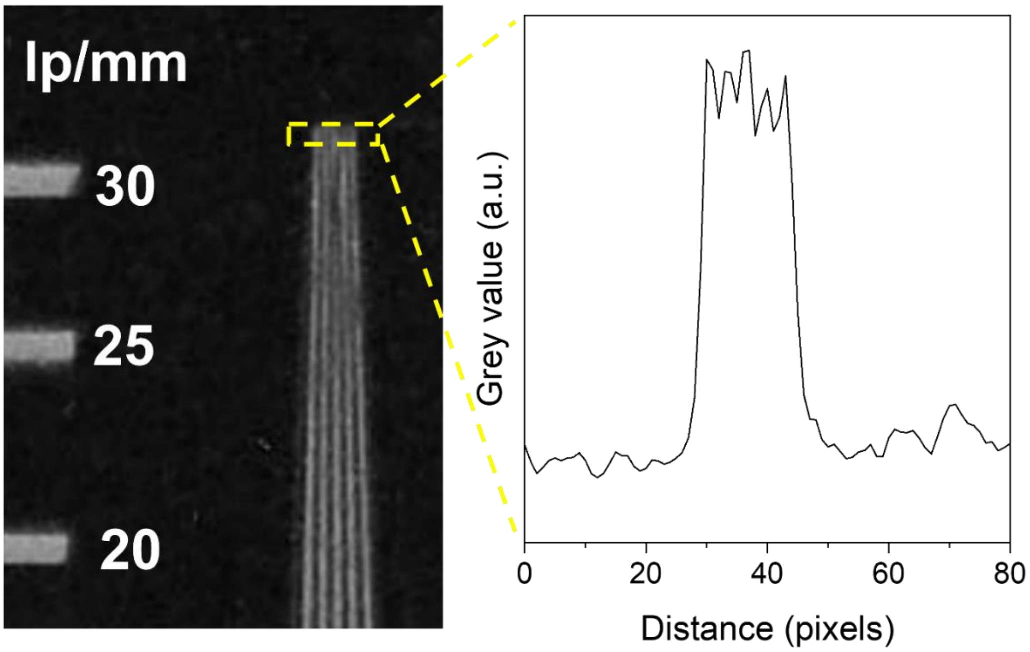

Figure S39. The spatial resolution data determined by a standard line-pair board (left) and the corresponding fitting of the intensity spread profile (right) of a $[Cu_4I_4(PPh_2Et)_4]$ glass with a thickness of 50 μm.

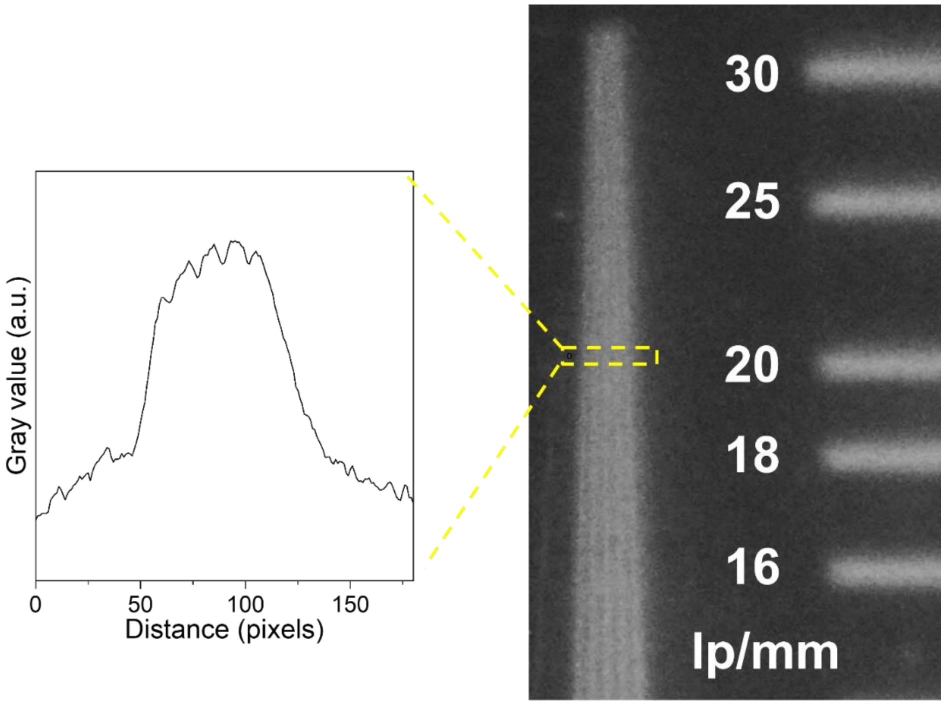

Figure S40. The spatial resolution data determined by a standard line-pair board (right) and the corresponding fitting of the intensity spread profile (left) of a $[Cu_4I_4(PPh_2Et)_4]$ glass with a thickness of 1 mm.

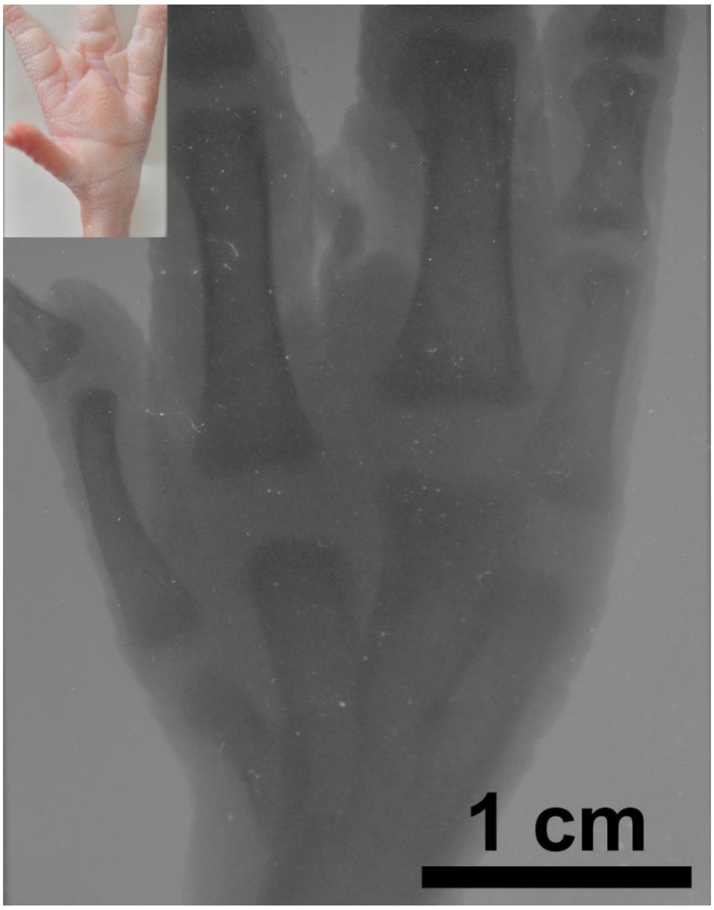

Figure S41. X-ray images of a chicken foot. Insets: the corresponding image under visible light.

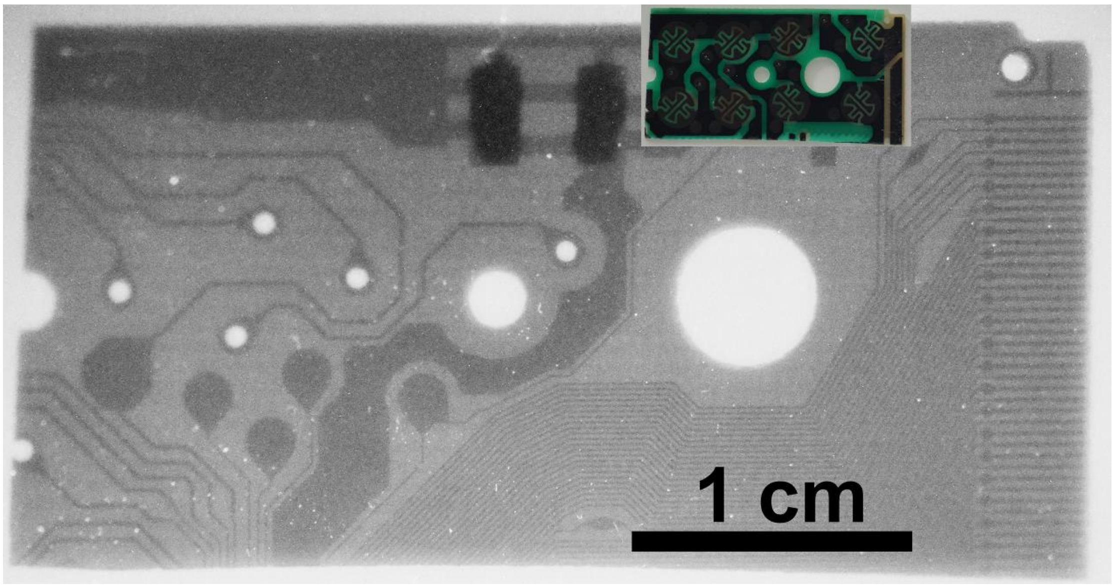

Figure S42. X-ray image of a circuit board. Insets: the corresponding image under visible light.

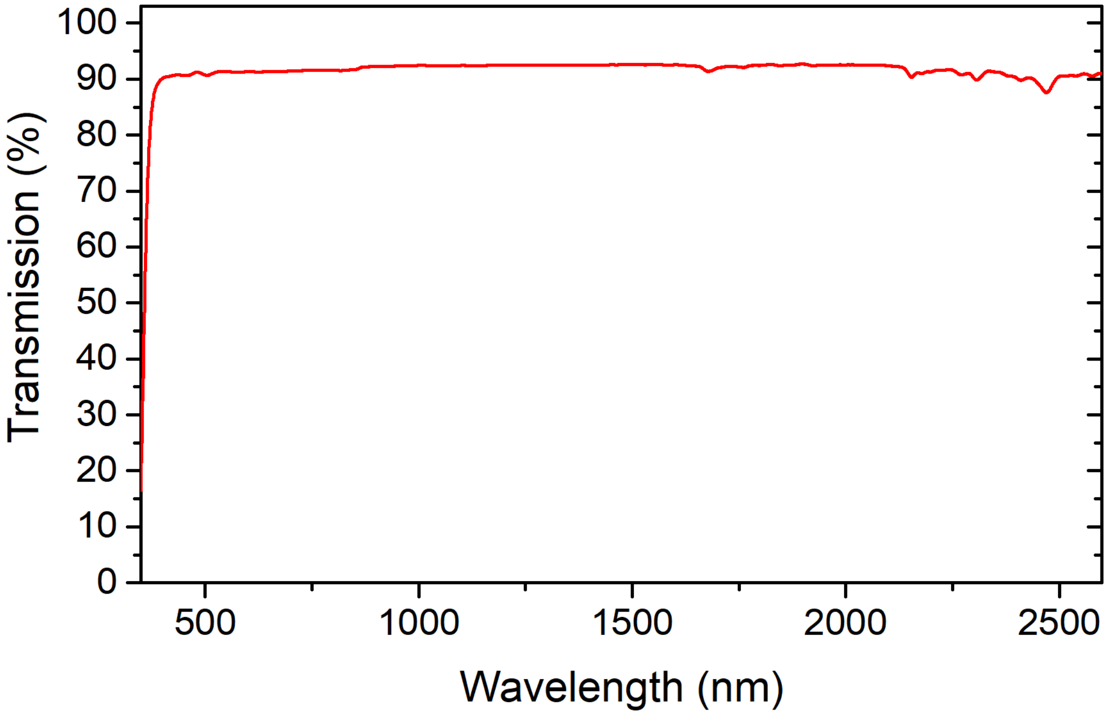

Figure S43. Transmission through [$Cu_4I_4(PPh_2Et)_4$] glass in near-infrared (NIR) wavelengths.

Video S1: AIMD simulation trajectory at 300 K, 600 K, 1000 K and 1200 K.

Video S2: AIMD simulation trajectory for the quenching process from 1000 K to 273 K

Video S3: Pulling glass fibers from amorphous phase.

Video S4: Waveguiding through hybrid glass fibers.
